# An Analysis on the Performance of a Mobile Platform with Gas Sensors for Real Time Victim Localization

**DOI:** 10.3390/s21062018

**Published:** 2021-03-12

**Authors:** Antonios Anyfantis, Spyridon Blionas

**Affiliations:** Systems Laboratory, Department of Informatics and Telecommunications, Faculty of Economics and Technology, University of Peloponnese, GR-221 31 Tripoli, Greece; aanyfantis@gmail.com

**Keywords:** air exchange, gas sensors, search and rescue, survivor detection

## Abstract

This work concerns the performance analysis of the sensors contained in a victim detection system. The system is a mobile platform with gas sensors utilized for real time victim localization in urban environments after a disaster has caused the entrapment of people in partially collapsed building structures. The operating principle of the platform is the sampling of air from potential survival spaces (voids) and the measurement of the sampled air’s temperature and concentration of CO_2_ and O_2_. Humans in a survival space are modelled as sources of CO_2_ and heat and sinks of O_2_. The physical openings of a survival space are modelled as sources of fresh air and sinks of the internal air. These sources and sinks dynamically affect the monitored properties of the air inside a survival space. In this paper, the effects of fresh air sources and internal air sinks are first examined in relation to local weather conditions. Then, the effect of human sources of CO_2_ and sinks of O_2_ in the space are examined. A model is formulated in order to reliably estimate the concentration of CO_2_ and O_2_ as a function of time for given reasonable entrapment scenarios. The input parameters are the local weather conditions, the openings of the survival space, and the number and type of entrapped humans. Three different tests successfully verified the presented theoretical estimations. A detection system with gas sensors of specified or measured capabilities, by utilizing this model and based on the expected concentrations, may inform the operator of the minimum required presence of humans in a survival space that can be detected after “some time”.

## 1. Introduction

The extraction of people from partially or completely destroyed buildings after a catastrophic event is executed in Urban Search and Rescue (USaR) missions. The catastrophic event may be a natural disaster such as a seismic event, an explosion due to equipment malfunction, or the result of a malicious act. Some of the people—victims—are confined in survivable voids under the building debris where the air refreshment rates are low. Canines trained for search and rescue are one of the established tools for victim localization, while a variety of technological systems have emerged intending to assist, complement, or even replace them [1]. The utilization of an exhaled air based human detection system with gas sensors in spaces where the rate of air refreshment is low is ideal. [2] This is because such operating parameters allow for the accumulation of measurable levels of human presence indicators. The operator of the victim detection system can locate a survivor by scanning the air similar to the way a search canine would.

In this work, an analysis and a methodology is proposed for the evaluation of the expected performance of a system with gas sensors in USaR missions, i.e., to evaluate with an estimated airflow rate the minimum ”size” of a victim which it is possible to detect This work uses established methods and models for calculating gas concentrations in building constructions [3,4,5,6] and applies them in the USaR field of research.

The presented analysis, because of its “generic” organization and presentation, is not limited to the exact sensor devices it is associated with in this work but can be applied to any air-based detection system operating in the described operating conditions. The analysis is not intended to be used in real time to actually detect human presence; the designed system with gas sensors will be used for that purpose. The analysis is used to first establish if a system that is envisioned has the potential to work without having to build and test a prototype. Thus, it serves as a tool to perform the feasibility analysis. After a detection system is implemented, the analysis provided in this work can be used to provide a quick estimate of the feasibility of detecting, in the USaR operations field, a human at a specific location with given weather conditions. The ideal tool is one that works anywhere. There are many human detection technologies, “orthogonal” to each other, which have advantages and limitations. Having a method to quickly select, or rather disqualify the usage of a tool and save precious time, is a step forward.

From the analysis carried out in this study, manufacturers of systems with gas sensors targeting the USaR rescue operations market will be able to improve their future systems by implementing the results of this work in their design. A future system with gas sensors could inform the operator of the estimated capabilities of the system by receiving the weather information (wind and temperature) and examining the void direction/position and its visible apertures. At present, miniature portable weather stations exist, which are capable of measuring temperature and wind speed. Optical based technologies also exist for creating 3D representations of large areas and structures and could be incorporated to automatically detect cavities and calculate opening sizes. This will enable users or future AI (Artificial Intelligence) based platforms to decide if a specific system with gas sensors is reliable enough to use and to prioritize the available search tools and not lose valuable time that may cost human lives. It would then become one of the technologies that can assist, complement, or even replace trained search and rescue dog, especially in confined and narrow places.

The human detection gas sensor-based scheme examined in this work operates by measuring CO_2_ and O_2_ concentration in the air in an attempt to detect changes caused by trapped humans. In atmospheric air CO_2_ and O_2_, have high background measurement levels, and they are emitted by human breath at substantially differentiated levels. Specifically, the CO_2_ concentration is approximately 400 ppm in the atmosphere and 46,000 ppm in exhaled human breath (Parts per Million, ppm). Concerning O_2_, atmospheric air has up to 20.946% and exhaled breath down to 16%. At a collapsed site’s cavity, when humans are trapped inside, it is expected that the levels of the CO_2_ and O_2_ concentrations will be affected since the humans are actually considered as sources of CO_2_ and sinks of O_2_, thus changing the composition of the air inside the void. The number of victims in a cavity and their weight, as well as their metabolic state, define the CO_2_ supply rate in the cavity (source), and the O_2_ absorption rate (sink). Additionally, the entire cavity where a victim is entrapped is not sealed. In general, it is expected to be ventilated, allowing the escape of target particles (CO_2_ and O_2_), and the renewal of the gas mixture inside the void with fresh atmospheric air. The ventilation of a cavity by outside air sources (fresh air or air from an adjacent cavity) is influenced by the openings of the cavity, by the local weather conditions, such as the wind speed and direction and by other airflows, such as thermal flows. For a human detection system with gas sensors to be efficient in rescue operations, a proper estimation of the concentrations of the target gases/substances to be detected in a specific cavity, is crucial. Various sensor technologies have different measurement resolutions that affect detection above the noise floor. The differentiation of the concentrations of CO_2_ and O_2_ inside the void, in comparison with those in the fresh atmospheric air just outside the void, should be above the noise floor, for it to be feasible to reveal information about the presence of victims inside a cavity under investigation. In this paper, a detection platform with sensors that perform similarly to those listed in Section 3.2 is implied.

Section 2 of the paper estimates the anticipated concentration of CO_2_ and O_2_ of a cavity as a function of time (in the rest of the paper, the terms void and cavity are used as equivalent). Section 3 examines various cases of entrapped humans in a void, the time needed for equilibrium for these various cases, and the anticipated performance of a system with gas sensors considering the characteristics of the CO_2_ and O_2_ sensors, and the noise floor for these gases. Section 4 studies the effect that weather conditions (external wind and temperature difference) have on the levels of CO_2_ and O_2_ equilibrium concentrations in voids with openings. Section 5 briefly presents the system with gas sensors and lists the selected sensors and various design decisions and assumption, while Section 6 presents the test results obtained with this system with gas sensors and compares the test results with the analysis made in Section 2, Section 3 and Section 4. Section 7 concludes the paper.

## 2. Estimation of Mean Concentrations of CO_2_ and O_2_ in a Cavity

The entire void is considered to be a confined space of constant volume V_c_. Its openings will be handled as sources of fresh atmospheric air and sinks of the cavity’s air simultaneously. Entrapped humans, similarly, will be seen as sources of exhaled air and sinks of the cavity’s air at the same time. Taking into account all the sources and sinks of air in a void, as well as the pressure and temperature of the air inside and outside that cavity, and assuming that the movement of air molecules inside creates a uniform distribution, then the mean concentrations of CO_2_ and O_2_ inside the void can be estimated. Even a rough approximation of CO_2_ and O_2_ mean concentrations inside the void, and then a comparison with the concentrations outside of that and with the sensors’ noise floor, are sufficient to make an initial decision about the value of investigating a void for the existence of entrapped humans. Specifically, the sampled and measured air by the system with gas sensors, in a cavity under investigation, and the differentiation of the concentrations of CO_2_ and O_2_ inside the void, compared to those of the air just outside it, must be higher than the system’s lower detection limits to be detectable.

The CO_2_ and O_2_ sources and sinks in a cavity must be studied in order to estimate their mean concentrations as a function of time. Concerning the initial conditions of the problem after the collapse of a building because of an abrupt catastrophic event, the composition of the atmospheric air inside the created voids/cavities in the rubble is assumed to be near the same composition as that of the atmospheric air outside them. In the case that there are entrapped humans in the void, the air composition and temperature will be affected. The first problem is to estimate how all these factors are combined and how they affect CO_2_ and O_2_ mean concentrations in the void, as a function of time.

The mean concentration C_x_ of CO_2_ or O_2_ ($C_{\left({\mathrm{CO}}_{2}\right)}$ or $C_{\left(O_{2}\right)},$ respectively), inside a room or a cavity of volume V_C_, is given in ppm by the formula $C_{x}=\frac{n_{x}}{n_{c}}$ ($n_{x}$ is the number of molecules of the specific gas x component in the total cavity, and $n_{c}$ the number of air molecules in the cavity). Since the cavity’s volume V_C_, remains unchanged, the total number of air molecules in the cavity for a specific temperature could also be considered as unchanged. In a cavity that has a number of openings for fresh atmospheric air to enter, and with entrapped humans, assuming that before the entrapment of the humans the space was filled with fresh atmospheric air, the parameters in Table 1 would influence the number of molecules of CO_2_ or O_2_ in the cavity:

The temperature difference between *T_c_* and *T_f_*, might increase or decrease the temperature in the cavity from *T_c_* to *T’_c_* and change the total number of air molecules in the cavity. Nevertheless, this change does not affect the concentration of any of the air components as far as the concentration expression refers to volume ratio (air component partial volume), or mole fraction/ratio (ppm or %). According to gas laws the proportionalities between the air components’ molecules, and thus their partial volumes, will remain the same. The temperature difference creates airflows that will influence the gas concentrations as shown in Section 4.2.

Assuming that when fresh air enters the void its temperature in a very short time becomes the same as the temperature inside the void, the principle used to estimate mean concentration of CO_2_ or O_2_ inside the cavity, is that the cavity’s volume and pressure, remain unchanged and thus airflows coming into the cavity must be balanced with the exhaust flows. Airflows are considered for a specific opening (humans are handled also as openings), as positive when they enter the cavity and negative when they exit.

Then:

(1) $$\sum F=0\Rightarrow F_{f}+F_{e}-F_{c}-F_{i}=0$$

Additionally, because the inhaled and exhaled air flow rates are the same ($F_{e}=F_{i}$), the result is $F_{f}=F_{c}$. Also, the concentration of gases exiting the cavity is the same with the concentration of gases inhaled by the victim so *C_xi_* = *C_xc_*. The change of the partial volume of an air component x in the cavity, within a very small fraction of time dt, it would be:

(2) $$\Delta V_{x}=\left(F_{f}C_{xf}+F_{e}C_{xe}-F_{c}C_{xc}-F_{i}C_{xc}\right)dt$$

Also, the concentration change is given by:

(3) $$dC_{xc}=\frac{\Delta V_{x}}{V_{c}}=\frac{\left(F_{f}C_{xf}+F_{e}C_{xe}-\left(F_{c}+F_{i}\right)C_{xc}\right)dt}{V_{c}}$$

As already mentioned, $F_{e}=F_{i}$ and $F_{f}=F_{c}$. Therefore, Equation (3) becomes:

$$\frac{dC_{xc}}{dt}=\frac{F_{f}C_{xf}+F_{e}C_{xe}-\left(F_{f}+F_{e}\right)C_{xc}}{V_{c}}\Rightarrow$$

(4) $$\frac{dC_{xc}}{dt}+\frac{\left(F_{f}+F_{e}\right)}{V_{c}}C_{xc}=\frac{F_{f}C_{xf}+F_{e}*C_{xe}}{V_{c}}$$

The solution of the differential Equation (4) is as follows:

(5) $$C_{xc}\left(t\right)=K+K_{0}e^{-\;\frac{F_{f}+F_{e}}{V_{c}}t}$$

Substitutig Equation (5) in (4), and by assuming that at time *t* = 0 (when the catastrophic event happened), $C_{xc}\left(0\right)=C_{xf}$, coefficients *K* and *K*_0_ are calculated and the (5) (the mean concentration of CO_2_ or O_2_ in the cavity, as a function of time), becomes:

(6) $$C_{xc}\left(t\right)=\frac{F_{f}C_{xf}+F_{e}C_{xe}}{F_{f}+F_{e}}+\left(C_{xf}-\frac{F_{f}C_{xf}+F_{e}C_{xe}}{F_{f}+F_{e}}\right)e^{-\;\frac{F_{f}+F_{e}}{V_{c}}t}$$

Equation (6) has the variables: time (*t*), exhaled air flow rate (*F_e_*), fresh air flow rate (*F_f_*), fresh air concentration of the x atmospheric air’s gas component (*C_xf_*), the concentration of the x exhaled air’s gas component (*C_xe_*), and the volume of the cavity (*V_c_*).

Verifying the above formula $C_{xc}\left(t\right)$, we get $C_{xc}\left(0\right)=C_{xf}$, as expected.

The variables of time (*t*) and of the cavity’s volume (*V_c_*) may be eliminated by examining (6) when t→∞, i.e., when $e^{-\;\frac{F_{f}+F_{e}}{V_{c}}t}\to 0$.

(7) $$lim_{t\to \infty }C_{xc}\left(t\right)=\frac{F_{f}C_{xf}+F_{e}C_{xe}}{F_{f}+F_{e}}$$

A specific system with gas sensors for USaR operations works by measuring the concentrations of CO_2_ and O_2_ in the fresh air ($C_{xf}$) at the entrance of a void and evaluating the standard deviation (SD) of the measurements of its sensors. It may be aware of the detection capabilities of each sensor by evaluating Equation (7) according to the other three variables, the expected airflow rates *F_f_* and *F_e_* and of the concentrations of CO_2_ and O_2_ in the exhaled air ($C_{xe}$). The next sections will examine the range of values for *F_e_*, *C_xe_* and *F_f_*.

## 3. Air Sources and Sinks Inside a Rubble Cavity and Anticipated Performance of the Gas Sensor-Based Detection System

In this, section we will first present the various cases of entrapped humans in a void and their expected levels of *F_e_* and *C_xe_*, and then the estimation of the standard deviation of the measurements of the gas sensors “noise floor”), the anticipated performance of a system with gas sensors considering the estimated standard deviation of the CO_2_ and O_2_ sensors of the system with gas sensors, and finally the time needed for equilibrium for various cases of entrapped humans and cavities sizes.

### 3.1. Expected Levels of Exhaled Air Flow Rate (F_e_) and Concentrations $C_{(CO_{2})e}$ and $C_{(O_{2})e}$

Exhaled air from the entrapped human(s), contains an increased concentration of CO_2_ (as part of human metabolic process), in comparison with the CO_2_ of the fresh atmospheric air outside the rubble. Specifically, exhaled air composition, according to literature, [7,8,9,10] is $C_{({\mathrm{CO}}_{2})e}\;\approx \;40.000–53.000\;\mathrm{ppm}$. Fluctuations are also significant depending on the person’s characteristics and physical condition [8].

Concerning O_2_, exhaled air contains decreased concentration levels in comparison to the O_2_ in the fresh atmospheric air. Exhaled air contains $C_{(O_{2})e}\approx 13.6\%–16.0\%$ [8,9,11], and dry air of normal atmospheric air, contains $C_{(O_{2})f}\approx 20.536\%–20.946\%$, [10,12].

The exhaled air flow rate for each victim depends on their weight. The number of breaths must be defined for a specific time period and the volume of the average breath of a person, in order to estimate the exhaled air flow rate. The flow rate of exhaled air $F_{e}$ in L/min, results as $F_{e}=n_{b}v_{t}$, where *F_e_* is the exhaled breath flow rate (in L/min), $n_{b}$ is the number of breaths in one minute (in # breaths/min), and $v_{t}$ tidal volume (in L). Specifically, the respiratory rate for adults is usually 12–20 breaths per minute [13,14]. Elderly people may have a slower breathing rate (10–18 breaths per minute) [14]. Children have a faster breathing frequency reaching up to 25–40 breaths per minute for infants [15], as shown in Table 2. 

The volume of the average breath (tidal volume) of an adult or elderly person is estimated between 390 (women) and 500 mL (men) [16]. Infants’ and children’s tidal volume is between 0.1 and 0.2 l (Table 2). However, in absolute terms, the amount of air a person needs is determined by the mass of the person. On average a person requires 7 mL or air per kg of body weight [16]. So an average male adult weighing 75 kg would have a tidal volume of 0.525 L, and as such, an exhaled air flow rate of $F_{e}=6.3-10.5\;L/\min$. In Table 2, the exhaled air flow rates for individuals at different ages [17,18,19] are calculated.

Table 2 gives a range for the flow rate of exhaled air $F_{e}$, in L/min, from $F_{e}=20\frac{\mathrm{breaths}}{\min}\frac{0.1\;l}{\mathrm{breath}}=\;2.0\;L/\min$, (in case of a child 3 years old), up to $F_{e}=20\frac{\mathrm{breaths}}{\min}\;0.525\frac{l}{\mathrm{breath}}=10.5\;L/\min$, (in case of an adult male)

The inspiration flow rate is considered the same as exhalation $F_{e}=F_{i}$, and the concentration of a cavity’s inhaled air in CO_2_ or O_2_ is denoted as $C_{({\mathrm{CO}}_{2})c}$ and $C_{(O_{2})c}$, respectively.

### 3.2. Gas Sensors’ Concentrations Measurements $C_{(CO_{2})c}$ and $C_{(O_{2})c}$ “Noise Floor”

Systems with gas sensors can use both high speed and low speed sensors. High speed sensors, such as non-dispersive infrared absorption sensors (for CO_2_), ensure a fast detection result, but have low accuracy (3–5%). Sensor technologies which exhibit a larger response time, like luminescence quenching by oxygen based (for O_2_), have higher precision (~2.7%) [20]. Fast sensors are preferable for systems with gas sensors targeted to USaR operations. The main reason is that they deliver data fast enough (up to 20 Hz), to detect a point of interest during rubble searches. On average, this is adequate to detect a change in concentration between 2 adjacent points in a cavity (about 1 cm distance for a robotic platform travelling at a speed up to 20 cm/s). Nevertheless, an accuracy of 5% on top of the unavoidable noise can further distort the signal and reduce the accuracy by up to 15%. There are alternatives adopted by such systems [20], by using either concurrently higher precision, but slower sensors to complement the weakness of the low accuracy of the high speed sensors or by using a pre-processing low-pass filter. In an attempt to evaluate CO_2_ and O_2_ sensors in a system with gas sensors [21], the noise of both sensors has been measured and shows that CO_2_ was in the range of 350–535 ppm (average 426 ppm), and O_2_ percentage range was between 20.67–20.8% (average = 20.724%). In dry normal atmospheric air, concentrations of CO_2_ and O_2_ are roughly $C_{({\mathrm{CO}}_{2})f}=405–420\;\mathrm{ppm}$, and $C_{(O_{2})f}\approx 20.536\%–20.946\%$, [10,12]. The statistical analysis for the measurements for both sensors is summed up in Table 3 (3600 measurements of fresh atmospheric air, in 1 h by the CO_2_ and O_2_ sensors):

The standard deviation of the CO_2_ sensor measurements is three orders of magnitude larger than that of O_2_. The O_2_ sensor is much more accurate and provides a much smoother and more stable output. In order for someone to be detected under the assumed conditions using the latest value acquired from each sensor, the values must be at least two standard deviations larger (CO_2_) or smaller (O_2_), from the mean values of fresh air’s concentrations. Taking into account the standard deviation estimated in Table 3, this specific system with gas sensors with the measurements presented here, can detect entrapped victims in a cavity, with enough certainty and without creating false alarms to the rescue teams, when the following inequality holds for the CO_2_ sensor:

(8) $$C_{({\mathrm{CO}}_{2})c}\geq \left(1+2stdev_{\bar{C_{({\mathrm{CO}}_{2})f}}}\right)\bar{C_{({\mathrm{CO}}_{2})f}}$$

$\bar{C_{({\mathrm{CO}}_{2})f}}$ is the mean value of a cavity’s fresh air concentration in CO_2_, and $stdev_{\bar{C_{({\mathrm{CO}}_{2})f}}}$ its standard deviation.

Similarly, for the O_2_ concentration should hold:

(9) $$C_{(O_{2})c}\leq \left(1-2stdev_{\bar{C_{(O_{2})f}}}\right)\bar{C_{({\mathrm{CO}}_{2})f}}$$

$\bar{C_{(O_{2})f}}$ is the mean value of a cavity’s fresh air concentration in O_2_, and $stdev_{\bar{C_{(O_{2})f}}}$ its standard deviation.

According to the measurements presented in Table 3, (8) and (9) become in this case $C_{({\mathrm{CO}}_{2})c}\geq 1.10798\bar{C_{({\mathrm{CO}}_{2})f}}$ and $C_{(O_{2})c}\leq 0.9995\bar{C_{(O_{2})f}}$. These threshold values are the worst-case scenario. If more than one measurement per sensor is used for decision making, particularly with filtering/averaging performed on a window of a number of measurements (filter size), the decision threshold can be substantially reduced. The trade-off when selecting a larger number of samples (measurements) to apply to the filter is the increased response time because of the filter delay and, consequently, the reduced search/scanning speed that can be achieved. Nevertheless, the search speed requirements in USaR operations are less than 0.1 cm/s and even a filter of length 240 would delay the response for less than a few centimeters.

### 3.3. Mean Concentrations $C_{(CO_{2})c}$ and $C_{(O_{2})c}$ in Equilibrium and Human Detection Limits

In Section 2, it is shown that for t→∞ the concentration of CO_2_ or O_2_ in a cavity/void $C_{xc}$ converges to, $C_{xc}=\frac{F_{f}C_{xf}+F_{e}C_{xe}}{F_{f}+F_{e}}$. In Section 3.1, the range of values of the CO_2_ and O_2_ concentrations in humans’ exhaled breath is presented and in Section 3.2 the range of values of the CO_2_ and O_2_ concentrations in the fresh atmospheric air. Comparing these concentrations we get respectively for their range of values, $C_{({\mathrm{CO}}_{2})e}\approx 95C_{({\mathrm{CO}}_{2})f}–130C_{({\mathrm{CO}}_{2})e}$, and $C_{(O_{2})e}\approx 0.65C_{(O_{2})f}–0.78C_{(O_{2})f}$. In Section 3.1 it is shown, also, that for an individual human $F_{e}$ ranges from $F_{e}=\;2.0\;L/\min$, up to $F_{e}=\;10.5\;L/\min$ and in Section 3.2, it is found that “alarm” CO_2_ and O_2_ concentrations are $C_{({\mathrm{CO}}_{2})c}\geq 1.10798*C_{({\mathrm{CO}}_{2})f}$ and $C_{(O_{2})c}\leq 0.9995*C_{(O_{2})f}$.

Applying (7), inequality (8) results for the CO_2_ sensor measurements in the following:

$$C_{({\mathrm{CO}}_{2})c}=\frac{F_{f}C_{({\mathrm{CO}}_{2})f}+F_{e}C_{({\mathrm{CO}}_{2})e}}{F_{f}+F_{e}}\geq 1.10798C_{({\mathrm{CO}}_{2})f}\Rightarrow$$

(10) $$\frac{F_{f}+F_{e}\frac{C_{({\mathrm{CO}}_{2})e}}{C_{({\mathrm{CO}}_{2})f}}}{F_{f}+F_{e}}\geq 1.10798$$

Similarly, by using (8), inequality (11) for O_2_ becomes:

$$C_{(O_{2})c}=\frac{F_{f}C_{(O_{2})f}+F_{e}C_{(O_{2})e}}{F_{f}+F_{e}}\leq 0.9995C_{(O_{2})f}\Rightarrow$$

(11) $$\frac{F_{f}+F_{e}\frac{C_{(O_{2})e}}{C_{(O_{2})f}}}{F_{f}+F_{e}}\leq 0.9995$$

Inequality (10) reveals that the “most difficult case” is when the concentration of CO_2_ in the exhaled breath is minimum ($C_{({\mathrm{CO}}_{2})e}\approx 40,000\;\mathrm{ppm}$) [7,8,9,10], and in the fresh air maximum ($C_{({\mathrm{CO}}_{2})f}=420\;\mathrm{ppm}$), therefore, $\frac{C_{({\mathrm{CO}}_{2})e}}{C_{({\mathrm{CO}}_{2})f}}\approx 95.238$, then to rely on the CO_2_ sensor measurements for human detection, the following inequality has to be true:

(12) $$F_{f}\leq 871.74F_{e}$$

This finding shows that in the case that the flow rate of fresh air is $F_{f}\geq 871.74\;F_{e}$, then this specific CO_2_ sensor of the system with gas sensors might not be able to detect a single victim with such characteristics (i.e., exhalation flow rate *F_e_*). For example, the system with gas sensors will not detect a child (3 years old) alone in a void, if $F_{f}\geq 2\;m^{3}/\min$, or an adult male if $F_{f}\geq 7\;m^{3}/\min.$

For O_2_, similarly, when the concentration of O_2_ in the exhaled breath is maximum ($C_{(O_{2})e}\approx 16\%$) [7,8,9,10], and minimum in fresh air ($C_{(O_{2})f}\approx 20.536\%$), thus $\frac{C_{(O_{2})e}}{C_{(O_{2})f}}\approx 0.779$, then the O_2_ sensor may detect a human only when for $F_{f}$ it holds:

(13) $$F_{f}\leq 440.76\;F_{e}$$

Inequality (13) shows that in the case that the flow rate of the fresh air *F_f_*, is $F_{f}\geq 440.76\;F_{e}$, the O_2_ sensor might not be able to detect a victim. Specifically, with a single measurement, if $F_{f}\geq 1.2\;m^{3}/\min$, a child (3 years old), alone in a void, might not be detected, and this is also true for an adult male alone in a void if $F_{f}\geq 4\;m^{3}/\min.$

Assuming that the rescue teams need to evaluate the probability of detecting humans in a cavity, under certain conditions (estimated fresh air flow rate from the cavity openings), then by using the presented inequalities, it is possible to define the “resolution” of the system with gas sensors, i.e., the capability level performance of the system with gas sensors under those specific conditions.

### 3.4. Mean Concentrations and Time Needed for Equilibrium (t→∞)

When t→∞, in (6) the term $e^{-\;\frac{F_{f}+F_{e}}{V_{c}}t}\to 0$ and it holds (7): $lim_{t\to \infty }C_{xc}\left(t\right)=\frac{F_{f}C_{xf}+F_{e}C_{xe}}{F_{f}+F_{e}}$

A reasonable approximation for $e^{-\;\frac{F_{f}+F_{e}}{V_{c}}t}\to 0$ and to reach an equilibrium of CO_2_ or O_2_ concentration in a cavity is when $\frac{F_{f}+F_{e}}{V_{c}}t\approx 5$ and $e^{-\;\frac{F_{f}+F_{e}}{V_{c}}t}\approx 0.0067$, (ignored as small enough). Consequently, when $t\approx 5\frac{V_{c}}{F_{f}+F_{e}}$, rescue teams may consider that equilibrium is reached and the systems with gas sensors may be used to detect entrapped humans.

By using the estimated detection limits of the system with gas sensors in Section 3.3, and assuming three different cases of a lone, entrapped human, in Table 4 the time needed for equilibrium in 3 different sized cavities with different capacities, 52.5 m^3^ (up to 15 victims), 26.5 m^3^ (up to 5 victims) and 10 m^3^ (up to 2 victims) is presented.

Analyzing the results presented in Table 4, it is revealed that within less than 4 h even in a large cavity where up to 15 people may fit, the system with gas sensors would be able to detect a child (3 years old), entrapped alone in a void, assuming that the weather conditions do not create a fresh air flow rate of more than 1.2 m^3^/min. Weather conditions and their influence on the flow rate of fresh air coming into the cavity are analyzed in Section 4.

## 4. Weather Conditions (External Wind and Temperature Difference), and Expected Fresh Atmospheric Air Flow Rate (*F_f_*) in Rubble Voids with Openings

The airflow rate $F_{f}$ of fresh atmospheric air towards the inside of the void and the airflow rate $F_{c}$ from inside the void to the outside ($F_{f\;}$=$\;F_{c}$), depend upon the areas and the resistances of the various apertures (openings), created randomly at the time of the building collapsing, and the pressure differences between the incoming flow paths and the outgoing ones. The openings of a rubble void allow a number of airflow paths to be created among the cavity’s ends that have different pressures. Pressure differences may be due either to wind and/or to differences in density of the internal and outside fresh air because of a temperature difference (stack effect). In addition, the surrounding fabric temperatures in a cavity and heating/cooling loads inside the cavity, as well as the existence of entrapped humans, influence the internal air temperature. Wind creates a higher pressure outside the rubble in comparison to the pressure inside the cavity. Stack effect also creates a pressure difference that depends on the temperature difference as will be explained further in this section. Incoming flow paths are created at the openings where outside total pressure (wind and stack effect), is higher than that inside when outgoing flows are formed at the openings where the inside pressure is higher than that outside. Varying wind speeds and directions will generate a fluctuating pressure difference and flow rates in voids near the border of a rubble pile because of the wind. Internal and external temperatures may also vary throughout the day and across the seasons and create variable flow rates in voids. Usually, annual wind speeds may span from 0–10 m/s in any of the 8 cardinal directions and the temperature may span from −10 °C to + 40 °C. This is the reason that the fresh incoming airflow rate is highly unpredictable since the prevailing weather conditions at the time of the disastrous event and the conditions in a cavity are not known. Pressure differences depend on a number of factors such as local wind speeds (outside of the rubble) and their corresponding “pressure coefficients” created at the openings of the void, the inside and outside (air) temperatures, the size and nature of the openings of the void, the nature of flow paths within the void, and the created flow regimes. There are many variables and parameters affecting these flow paths and regimes but, even rough approximations are sufficient for the targeted application.

We will study all cases of wind and temperature differences between the inside and the outside of a void, i.e., positive, negative, and for both of these, low and high differences, to address all possible wind and temperature-driven pressure differences and created flows in a void [3]. When both cases apply (wind and stack effect), then the air flowing into a void will vary depending on the relative strengths of the forces of the two effects and whether they act in the same or opposite direction [4].

The effect of the aforementioned factors will be further analyzed in order to estimate the range of values for the fresh air flow rate F_f_, into a cavity and its influence on the human detection capability of a system with gas sensors.

### 4.1. Wind Effect and Estimation of the Created Fresh Atmospheric Air Flow Rate (F_f_)

Wind outside the rubble drives air into the void through the openings on the windward side of the cavity where the surface pressure is high. The wind’s deflection on the upwind face of the rubble induces a positive pressure on it. The air flow then separates, resulting (most times) in negative pressure regions developed along the other sides of the rubble. A negative pressure distribution usually develops along the leeward, or upper side. Fresh air then passes from the one side of the void to the other side and exits through apertures on the leeward, or upper side, where lower pressure is created, and a flow path is formed. The higher the wind speed, the higher will be the incoming fresh air flow rate (*F_f_*) and the outgoing cavity air flow rate (*F_c_*) because of the wind effect (*F_f_* = *F_c_*).

As already mentioned, the pattern of pressure distribution arising from the parameters of a rubble cavity is extremely complex and considerable simplification is necessary in any mathematical representation.

Relatively to the static pressure of the free wind, the time-averaged pressure acting on any point “i” on the surface of a rubble may be represented by the following equation [3]:

(14) $$P_{W_{f_{i}}}=0.5\rho_{f}C_{p_{i}}v_{z}^{2}$$

where $P_{W_{f_{i}}}=$ surface pressure due to wind (Pa), $\rho_{f\;}$= density of fresh air (≈1.2754 kg/m^3^), $C_{p_{i}}$ is the wind pressure coefficient at a given position on the rubble surface (generally independent of the wind speed), and *v_z_* is the mean wind velocity at height *z* (m). The wind pressure coefficient, $C_{p_{i}}$, is a function of wind direction and of spatial position on the rubble surface. However, an accurate evaluation of this parameter is extremely difficult and normally involves wind tunnel tests using a scale model of the cavity and its surroundings (rubble). Wind pressure coefficient could be expressed as a single average value for each “face” of the rubble. Average values for buildings of simple shape in wind exposed locations are presented in [3], without taking into account the surrounding obstructions in shielding the building from wind. A synthesis of facade-averaged values for buildings subjected to varying degrees of shelter and wind directions is given in [5]. In that work, various cases of square plan buildings are presented of heights up to three stories, and the associated wind pressure coefficients range from $C_{p_{i}}\;$= 0.42 to 0.72 for the surface of the void that “directly” faces the wind. The averaged data provide a useful approximation for the rubble cases that rarely exceed three stories in height and are expected to be in suburban areas. In any case, our interest is to roughly estimate the values of incoming fresh airflow rate F_f_, or outgoing cavity air *F_c_* (*F_f_* = *F_c_*), and not to calculate accurate values. The wind speed mentioned in (14), uses the wind speed *v_z_* for a building of height *z* raised to the power of two. Wind statistics [22] show that a mean wind speed ($v_{ref}$), of 10 m/s, covers most of the cases of western countries meteorological data, even for extreme weather conditions. The final wind speed at the location of the rubble is a function of the local environment (topography, ground roughness, and nearby obstacles). The formula that calculates the final wind speed is:

(15) $$v_{z}=v_{ref}C_{R}C_{T}$$

where $v_{z}\;$= wind speed at height *z* (m/s), $v_{ref}$ = reference regional wind speed (m/s), roughness coefficient at height z, $C_{R}\left(z\right)=K_{R}\;\ln\frac{z}{z_{0}}$, with $K_{R}$ to be the terrain factor ranging for suburban to urban areas from 0.22–0.24, *z*_0_ is the roughness length in meters ranging for suburban to urban areas from 0.3 to 1 m, and $C_{T}$ = topography coefficient. Assuming that height *z* of the rubble ranges for suburban to urban areas from 5 to 10 m, the roughness coefficient at height *z*$,\;\;C_{R}\left(z\right)$ may range from 0.35 to 0.84. The topography coefficient $C_{T}$ accounts for the increase in the mean wind speed over isolated hills and escarpments and ranges from 1 (Urban/Suburban terrain) to 1.6 (isolated hills and escarpments). Taking into account all the above, the final speed $v_{z}$ of the wind is expected to range between 0.35 *v_ref_* and 0.84 *v_ref_*, for urban/suburban areas [5].

In case of an opening “*j*” on a void’s surface that is not facing the wind “directly”, the pressure is given by (14) as $P_{W_{f_{j}}}=0.5\rho_{f}C_{p}{}_{j}v_{z}^{2}$. Wind pressure coefficient ${C_{p}}_{j}$ ranges from ${C_{p}}_{j}\;$= −0.02 to −0.79 [5].

Then, considering the created airflow path “*i*–*j*” between two ends “*i*” and “*j*” of a void because of their total pressure difference $\Delta {p_{W_{f}}}_{“i-j”}={P_{W_{f}}}_{i}-{P_{W_{f}}}_{j}$ for a cavity in a rubble pile located in an urban/suburban location, by using (14) and (15), it becomes:

(16) $$\Delta {p_{W_{f}}}_{“i-j”}=0.5\rho_{f}\left(C_{p_{i}}-C_{p_{j}}\right){\left(C_{R}v_{ref}\right)}^{2}$$

For calculating the range of values of the pressure difference $\Delta {p_{W_{f}}}_{“i-j”}$ between two openings “*i*–*j*” we considered: (a) rubble height (no more than 10 m -about 3 building floors height-), (b) for urban/suburban surrounding terrain *v_z_* =$\;C_{R}v_{ref}\;$= 0.35 *v_ref_* – 0.84 *v_ref_* (v_ref_ is weather reported wind speed at a near to the rubble open country location), (c) for the opening “*i*” on the surface of the void that faces the wind $C_{p_{i}}=$ 0.42 to 0.72, and for the opening “*j*” on any other surface $C_{p_{j}}=\;$−0.02 to −0.79). In Table 5 we calculated for various “reference” wind speeds (*v_ref_*), the range of values for ${P_{W_{f}}}_{i}$ and ${P_{W_{f}}}_{j}$ and then by using those ranges we roughly estimated the expected minimum and maximum value of the pressure difference $\Delta {p_{W_{f}}}_{“i-j”}$ between of the two openings “*i*” and “*j*” of a rubble void, for various “reference” wind speeds.

The almost twentyfold difference between minimum and maximum expected $\Delta {p_{W_{f}}}_{“i-j”}$ is because of the large variation in the terrain (urban-suburban), and the spatial position of an opening on the rubble surface. Considering that the above estimations were made with the assumption that the wind is perpendicular to the surface of the opening ‘i”, it is evident that it is very improbable to encounter the maximum $\Delta {p_{W_{f}}}_{“i-j”}$ values.

Consequently, the specific airflow path, “*i*–*j*” will provide a total airflow rate of fresh air into the void, $F_{W_{“i-j”}}$ because of the total pressure difference $\Delta {p_{W_{f}}}_{“i-j”}$ across it, caused by wind. Then, to calculate $F_{{\left(W+S\right)}_{“i-j”}}$ in Pa, we applied the common orifice Bernoulli’s equation. To estimate the airflow rate $F_{W_{f}{}_{“i-j”}}$ we considered large openings of “window” size, so to allow air passing through the cavity without much kinetic energy dissipation. Large openings of window size are orifice type openings and the equation becomes the common orifice Bernoulli Equation [23]:

(17) $$F_{W_{f}{}_{“i-j”}}=C_{d}{}_{i}A_{i}\sqrt{\frac{2\Delta {p_{W_{f}}}_{“i-j”}}{\rho_{f}}}$$

$F_{W_{f}{}_{“i-j”}}$= volumetric flow rate through a large opening *i* (m^3^/s), $C_{d}{}_{i}\approx 0.61$ (Discharge coefficient), $A_{i}$ = the area of the opening “*i*” on the surface of the void that “directly” faces the wind (m^2^), $\;\rho_{f}$ = density of air (kg/m).

We applied (17) for such large openings on the front surface of the cavity that “directly” faces the wind, assuming that the air density is *ρ_f_* ≈ 1.2754 Kg/m^3^, and by using the minimum and maximum values of $\Delta {p_{W_{f}}}_{“i-j”}$ from Table 5 for some of the wind speeds (*v_ref_*), we calculated in Table 6 (urban/suburban terrain) for a child (3 years old) and an adult man the maximum area *A*_*i*_ that would satisfy (12) and (13), i.e., CO_2_ and O_2_ sensors respectively. Thus, Table 6 shows for indicative cases of wind speeds, for both sensors of the system with gas sensors (CO_2_ and O_2_), and for both types of victims, what the maximum opening area *A*_*i*_ is that allows a victim to be detectable.

For most wind speeds, in an Urban/Suburban terrain, as shown in Table 6, a system with gas sensors will detect humans of any age and gender group, even for the maximum expected $\Delta {p_{W_{f}}}_{“i-j”}$. In the extreme case of 10 m/s wind speed perpendicular to the surface of the void opening and for maximum $\Delta {p_{W_{f}}}_{“i-j”}$, a child (3 years old), might be difficult to be detected by both CO_2_ and O_2_ sensors, if the size of the openings is more than 6 cm × 6 cm.

### 4.2. Temperature Effect (Stack Effect)

Stack effect is the air flow rate of the outside fresh atmospheric air into a cavity (*F_f_*), driven by a temperature difference Δ*Τ* (Δ*T* = *T_f_* − *T_c_*) between the outside and the inside of the cavity.

The difference between outdoor and indoor temperatures during the year and during the day vary significantly. The outdoor environmental temperature depends mainly on the season of the year, the geographical location of the place, and the surrounding environment (rural, urban, suburban, etc.) [24,25]. The more urbanization, the higher the outdoor temperature [25]. Concerning indoor temperatures in urban environments and their difference with the nearby outdoor temperatures, they are affected by the type of the building construction, the surrounding vegetation, and the distance from the city center. Indoor temperatures in heated buildings during the winter can sometimes be much higher than outdoors (e.g., 20 °C higher), but even if the building is not heated, the indoor temperature can be up to 10 °C higher than outdoors. Temperature difference between indoors and outdoors varies also by the time of the day. Diurnal temperature range (DTR) is the difference between the highest and lowest temperature in the same day and both indoor and outdoor temperatures have a similar pattern of DTR in summer or winter if the building is not heated. Temperature difference between the indoor and outdoor environment is higher early in the morning (e.g., 8 °C), and lower in the evening (e.g., 3 °C), with indoor temperature always being higher than the outdoor, with the exception of extremely hot days in summer when outdoors it might be 1–2 °C higher in case of buildings with poor insulation [26,27,28]. Consequently, the most probable, when comparing indoor and outdoor temperature, is that the temperature *T_f_* of the external fresh air is lower than the indoor temperature in any kind of building.

At the time of a catastrophic event that could create cavities in a pile of rubble with entrapped humans, because of the abrupt conditions of the catastrophic event, it is assumed that fresh atmospheric air will be mixed with the indoor air in the created voids/cavities and their temperature *T_c_*, will be higher than that of the fresh air *T_f_*, and lower than the indoor temperature *T_i_* before the catastrophic event (*T_f_* < *T_c_*< *T_i_*). The only exception where we could have another relationship between temperatures would be an extremely hot summer day around noon time when the outdoor temperature *T_f_* could be 1–3 °C higher than the indoor *T_i_*. The temperature inside a cavity in that case would be higher than the outdoor temperature (*T_c_* > *T_f_*). Nevertheless, this is considered an extreme case. Thus, immediately after the creation of a rubble void with entrapped humans, it is most probable that the air temperature *T_c_* inside the void, will have a temperature difference of several degrees, expected to range between Δ*T* ≈ 1–10 °C, which creates enough pressure difference to create forces higher than the friction of the air when moving through the openings of the void, and thus the stack effect brings fresh atmospheric air into the cavity. In the cavity, heat losses from the building’s materials and from the human body [29] (radiative, convective, conductive, respiratory, and evaporative) increase the temperature of the colder fresh air that enters the void and a continuous airflow rate is maintained. Because of that airflow, the temperature inside the void decreases steadily, but very slowly. The temperature difference Δ*Τ* between outdoor temperature *T_f_* and the cavity’s temperature T_c_, decreases as well (Δ*T* = *T_f_* − *T_c_*).

The pressure difference because of temperature difference (stack effect), occurs because cooler outdoor air is denser than the warm air inside the cavity and consequently the hydrostatic pressure is different. Actually, gravity is the reason for the stack effect and the created pressure difference. The cold air from outside (of temperature *T_f_*), is heavier and with higher pressure and it enters through low positioned gaps and openings (e.g., “*i*”), into the cavity that has warmer, lighter and lower pressure air. The displaced air inside (of temperature *T_c_*), escapes through gaps and openings (e.g., “*j*”), at a higher level in the fairly large rubble voids (sometimes 2–3 m height). This direction of movement could be reversed in the rare case of a very hot summer day, around noon time as mentioned already, when the external air temperature could be 1–3 °C higher than the indoor temperature, and thus it may result in a lower temperature in the cavity than the outdoor temperature (*T_c_* < *T_f_*), and the colder air from the void will escape through low positioned gaps and openings, and warm air from outside will enter the void to balance pressure.

To estimate the stack effect’s air flow rate, when void openings are “window” sized, we applied the Bernoulli model. The volumetric flow rate through an opening “*i*” because of the stack effect, depends on the pressure drop at the opening “*i*” $\Delta p_{S_{i}}={P_{S_{f}}}_{i}-{P_{S_{c}}}_{i}$ (${P_{S_{f}}}_{i}$ is the “hydrostatic” atmospheric pressure of the outside fresh atmospheric air and ${P_{S_{c}}}_{i}$ is the “hydrostatic” atmospheric pressure of the air in the cavity, both at the opening “*i*”, and in Pa). $\Delta p_{S_{i}}$ is always positive (higher outdoor pressure) and, as a result, the fresh atmospheric air enters into the cavity, except in the rare case of a hot summer day in the afternoon [26,27,28]. At the other opening “*j*” at a higher level than the opening “*i*”, the pressure difference between the outdoor atmospheric air and of the air inside the cavity is $\Delta p_{S_{j}}={P_{S_{f}}}_{j}-{P_{S_{c}}}_{j}$ (${P_{S_{f}}}_{j}$ is the “hydrostatic” atmospheric pressure of the outside fresh atmospheric air, and ${P_{S_{c}}}_{j}$ is the atmospheric pressure of the air inside the cavity, both at opening “j”, in Pa). $\Delta p_{S_{j}}$ is negative because the warm air inside the cavity at the opening “*j*” has higher pressure ${P_{S_{c}}}_{j}$ than the atmospheric pressure ${P_{S_{f}}}_{j}$ outside of the void at the upper level of opening “*j*” and air goes out of the cavity. We define $\Delta p_{S_{fc_{“i-j”}}}$ as the total pressure difference between the two openings “*i*” and “*j*” $\Delta p_{S_{fc_{“i-j”}}}=\Delta p_{S_{i}}-\Delta p_{S_{j}}$. As shown in [6] $\Delta p_{S_{fc_{“i-j”}}}$ is given in Pa by:

(18) $$\Delta p_{S_{fc_{“i-j”}}}=\left(\rho_{f}-\rho_{C}\right)gz_{“i-j”}$$

The quantity $z_{“i-j”}$ represents the vertical distance between the “*i*” and “*j*” openings, *ρ_f_* and *ρ_C_* are the air density outside and inside the cavity, respectively, and *g* is the gravitational acceleration. This total pressure difference $\Delta p_{S_{fc_{“i-j”}}}$ creates an airflow between the two openings “*i*” and “*j*” that enters the void from the lower opening “*i*” and exits from the higher opening “*j*”. In the rare case of a hot summer’s day in the afternoon, the airflow has the opposite direction [26,27,28]. The rate $F_{fc_{S_{“i-j”}}}$ of this airflow is given by:

(19) $$F_{fc_{S_{“i-j”}}}=C_{d_{“i-j”}}A_{i}\sqrt{\frac{2\left|\Delta p_{S_{fc_{“i-j”}}}\right|}{\rho }}$$

$F_{fc_{S_{“i-j”}}}$ is the airflow rate through the openings “*i*” and “*j*” (m^3^/s), $C_{d_{“i-j”}}$ is the discharge coefficient for the airflow, **A*_*i*_* is the area of the opening “*i*” (m^2^), $\Delta p_{S_{fc_{“i-j”}}}$ is the total pressure difference because of the stack effect (Pa) and ρ is the air density (kg/m^3^).

Particularly for the discharge coefficient, it quantifies the airflow efficiency of a created airflow because of the stack effect, or alternatively, the airflow resistance of it and its values are 0.6 ± 0.1 and it varies according to the opening’s aspect ratio, direction/angle, pressure difference and temperature difference.

Some indicative values for a pressure difference created by a temperature difference of 10 °C (*T_f_* = 5 °C and *T_c_* = 15 °C), for a cavity 1 m high is 0.43 Pa and for 3 m 1.29 Pa [6]. When a temperature difference of 10 °C is between *T_f_* = 20 °C and *T_c_* = 30 °C, for a cavity 1 m high the pressure difference is 0.39 Pa and for 3 m high 1.17 Pa [6]. For a temperature difference of 10 °C between *T_f_* = −5 °C and *T_c_* = 5 °C, for a cavity 1 m high is 0.46 Pa and for 3 m high 1.39 Pa [6]. For a temperature difference of 5 °C between *T_f_* = 0 °C and *T_c_* = 5 °C, for a cavity 1 m high is 0.23 Pa and for 3 m high 0.68 Pa [6].

Generalizing (19) for many openings, then the total incoming air flow rate would be:

(20) $$F_{fc_{S_{sum}}}=\underset{n}{\sum }F_{fc_{S_{n}}}$$

$F_{fc_{S_{n}}}$ is the stack effect’s created airflow rate for each pair of openings given in (19). We approximated the stack effect’s total created airflow rate by making several assumptions. The first assumption was that all incoming openings are at a lower level of the void and that the outgoing ones at the top of the void are at height z. Then, the stack effect incoming air flow rate $F_{fc_{S_{sum}}}$ in (20) becomes:

(21) $$F_{fc_{S_{sum}}}\approx \underset{n}{\sum }C_{d_{n}}A_{n}\sqrt{\frac{2\left|\left(\rho_{f}-\rho_{C}\right)gz\right|}{\rho }}$$

*ρ* = *ρ_f_* when the temperature outside is lower than inside otherwise *ρ* = *ρ_C_*, $C_{d_{n}}$ and $A_{n}$ are the discharge coefficient and the area respectively for each pair of openings. Then further approximating $F_{fc_{S_{sum}}}$, we assumed that cavities, even those of large size (50 m^3^), have a lower height than 2–3 m, and *ρ_c_* is uniform inside the cavity and does not change because of the altitude. In [4] it is shown that when T_f_ < T_C_ then $\frac{\rho_{f}-\rho_{C}}{\rho_{f}}=\frac{T_{C}-T_{f}}{T_{f}+273}$, and in the very rare case of *T_f_* > *T_C_* then $\frac{\rho_{C}-\rho_{f}}{\rho_{C}}=\frac{T_{f}-T_{C}}{T_{C}+273}$ (*T_f_*, *T_c_* = temperature in Celsius degrees). Also, we assumed that the average value of the discharge coefficients for all openings is 0.61 and the total area of all openings is *A_sum_*, thus $\underset{n}{\sum }C_{d_{n}}A_{n}\approx 0.61A_{sum}$. Then, the approximated total incoming air flow rate in (21) becomes:

(22) $$F_{fc_{S_{sum}}}\approx 0.61A_{sum}\sqrt{\frac{2\left|\left(T_{C}-T_{f}\right)gz\right|}{T_{f}+273}}$$

Obviously, in the rare case that *T_f_* > *T_C_* then the outgoing air flow rate from the void it holds that:

(23) $$F_{fc_{S_{sum}}}\approx 0.61A_{sum}\sqrt{\frac{2\left|\left(T_{f}-T_{c}\right)gz\right|}{T_{c}+273}}$$

Applying (22), for various values of *z* (*z* = 1 m, 2 m and 3 m) and temperature differences ($T_{C}-T_{f}\;$= 3, 5, 10, and 15 °C), for both sensors of the system with gas sensors (CO_2_ and O_2_), and for both types of victims (a 3 year old child and an adult male), we calculated what is the maximum opening area *A*_*i*_ that would satisfy (12) and (13), i.e., CO_2_ and O_2_ sensors respectively and would allow a victim to be detectable by both the CO_2_ and O_2_ sensors of a system with gas sensors. Table 7 summaries the results.

For the stack effect, as shown from the calculations in Table 7, even for the rare case of a void 3 m high and with a temperature difference between outdoor temperature and void temperature of 15 °C, a system with gas sensors with sensor characteristics similar to that presented in Section 3.2 and in [20], and [21], would detect an entrapped 3-year-old child in a void with openings less than 0.3 m^2^ or else 50 cm × 50 cm wide (for an adult man it is more than 1 m^2^). In the extreme case of a very hot summer day around noon, because the temperature difference is expected to be less than 3 °C, a 3-year-old child could be detected even if the openings in a void of 3 m height have an area of more than 70 cm × 70 cm wide (~0.5 m^2^). A CO_2_ sensor alone could detect a 3-year-old child in a void 3 m high and with openings occupying a total area of more than half square meter (70 cm × 70 cm) in a temperature difference of more than 10 °C.

### 4.3. Stack and Wind Effect Combined

It is very rare for the stack effect and the wind effect not to act simultaneously. Even if they don’t, it would be for a very limited time [4]. We assume for a specific cavity an opening “*i*” providing an incoming airflow rate $F_{f}$ of fresh atmospheric air, because of the total pressure difference $\Delta p_{{\left(W+S\right)}_{i}}$ that is given by:

(24) $$\Delta p_{{\left(W+S\right)}_{i}}=P_{W_{f_{i}}}+\Delta p_{S_{i}}$$

$P_{W_{f_{i}}}$ is the pressure of the outside fresh atmospheric air because of wind, and $\Delta p_{S_{i}}$ is the pressure difference because of the stack effect at the opening “*i*”, as defined in Section 4.2 ($\Delta p_{S_{i}}={P_{S_{f}}}_{i}-{P_{S_{c}}}_{i}$, ${P_{S_{f}}}_{i}$ is the “hydrostatic” atmospheric pressure of the outside fresh atmospheric air and ${P_{S_{c}}}_{i}$ is the “hydrostatic” atmospheric pressure of the air in the cavity, all at the opening “*i*”, in Pa).

Also, we consider another opening “*j*” that provides an outgoing (from the cavity), airflow rate $F_{c}$ ($F_{f\;}$=$\;F_{c}$), because of a negative pressure difference between the atmospheric air outside of the cavity and the air inside the cavity, that drives air outside of the cavity and is given by:

(25) $$\Delta p_{{\left(W+S\right)}_{j}}=P_{W_{f_{j}}}+\Delta p_{S_{j}}$$

$P_{W_{f_{j}}}$ is the pressure of the outside fresh atmospheric air because of the wind, $\Delta p_{S_{j}}$ is the pressure difference because of the stack effect at the opening “*j*” as defined in Section 4.2 ($\Delta p_{S_{j}}={P_{S_{f}}}_{j}-{P_{S_{c}}}_{j}$, ${P_{S_{c}}}_{j}$ is the “hydrostatic” atmospheric pressure of the outside fresh atmospheric air and ${P_{S_{f}}}_{j}$ is the “hydrostatic” atmospheric pressure of the outside fresh atmospheric air, all at opening “*j*” in Pa).

Then, considering the created airflow path “*i*–*j*” between the two ends of the void because of their total pressure difference $\Delta p_{{\left(W+S\right)}_{“i-j”}}=\Delta p_{{\left(W+S\right)}_{i}}-\Delta p_{{\left(W+S\right)}_{j}}$, by combining (24) and (25) $\Delta p_{{\left(W+S\right)}_{“i-j”}}$ is given by:

(26) $$\Delta p_{{\left(W+S\right)}_{“i-j”}}=\left(P_{W_{f_{i}}}+\Delta p_{S_{i}}\right)-\left(P_{W_{f_{j}}}+\Delta p_{S_{j}}\right)=\Delta {p_{W_{f}}}_{“i-j”}+\Delta p_{S_{fc_{“i-j”}}}$$

$\Delta {p_{W_{f}}}_{“i-j”}$, according to the definitions in Section 4.1, is the pressure difference between the “*i*” and “*j*” openings because of wind ($\Delta {p_{W_{f}}}_{“i-j”}=P_{W_{f_{i}}}-P_{W_{f_{j}}}$) and $\Delta p_{S_{fc_{“i-j”}}}$ according to the definitions in Section 4.2 is the total pressure difference between the two openings “*i*” and “*j*” because of stack effect ($\Delta p_{S_{fc_{“i-j”}}}=\Delta p_{S_{i}}-\Delta p_{S_{j}}$).

Combining (16) and (18), (26) is modified as follows:

(27) $$\Delta p_{{\left(W+S\right)}_{“i-j”}}=0.5\rho_{f}\left(C_{p_{i}}-C_{p_{j}}\right){\left(C_{R}v_{ref}\right)}^{2}+\left(\rho_{f}-\rho_{C}\right)gz_{“i-j”}$$

Consequently, the specific airflow path, “*i*–*j*” will provide a total airflow rate of fresh air into the void, $F_{{\left(W+S\right)}_{“i-j”}}$ because of the total pressure difference $\Delta p_{{\left(W+S\right)}_{“i-j”}}$ across it caused by both wind and stack effect. Then, to calculate $F_{{\left(W+S\right)}_{“i-j”}}$ in Pa, we applied the common orifice Bernoulli’s equation [23]:

(28) $$F_{{\left(W+S\right)}_{“i-j”}}=C_{d_{“i-j”}}A_{i}\sqrt{\frac{2\Delta p_{{\left(W+S\right)}_{“i-j”}}}{\rho_{f}}}$$

$C_{d_{“i-j”}}$ is the discharge coefficient for the airflow that quantifies the efficiency of an opening (airflow resistance), $A_{i}$ is the area of opening “*i*” in m^2^. We assume that opening “*j*” is higher than the opening “*i*” whose inlet is situated in the external wind flow and that the external wind flowing around the opening “*i*” does not affect the discharge coefficient $C_{d_{“i-j”}}$ of the opening when the wind effect is combined with the stack effect.

By applying (27) we modified (28):

(29) $$F_{{\left(W+S\right)}_{“i-j”}}=C_{d_{“i-j”}}A_{i}\sqrt{\frac{2\left(0.5\rho_{f}\left(C_{p_{i}}-C_{p_{j}}\right){\left(C_{R}v_{ref}\right)}^{2}+\left(\rho_{f}-\rho_{C}\right)*g*z{}_{“i-j”}\right)}{\rho_{f}}}$$

As mentioned in Section 4.2, air densities are related to temperatures $\frac{\rho_{f}-\rho_{C}}{\rho_{f}}=\frac{T_{f}-T_{C}}{T_{f}+273}$ and (29) was re-formed to:

(30) $$F_{{\left(W+S\right)}_{“i-j”}}=C_{d_{“i-j”}}A_{i}\sqrt{\left(C_{p_{i}}-C_{p_{j}}\right){\left(C_{R}v_{ref}\right)}^{2}+2\frac{\left(T_{c}-T_{f}\right)gz_{“i-j”}}{T_{c}+273}}$$

In the rare case of a hot summer afternoon with combined, but opposing, wind and stack effect, the total airflow $HdF_{{\left(W+S\right)}_{“i-j”}}$ is given by:

(31) $$HdF_{{\left(W+S\right)}_{“i-j”}}=C_{d_{“i-j”}}A_{i}\sqrt{\left(C_{p_{i}}-C_{p_{j}}\right){\left(C_{R}v_{ref}\right)}^{2}-2\frac{\left(T_{c}-T_{f}\right)gz_{“i-j”}}{T_{c}+273}}$$

Generalizing (30) for many openings, then the total incoming air flow rate would be:

(32) $$F_{{\left(W+S\right)}_{sum}}=\underset{n}{\sum }F_{{\left(W+S\right)}_{n}}$$

$F_{{\left(W+S\right)}_{n}}$ is the wind and stack effects’ combined created airflow rate for each pair of openings given in (30). We also approximated here the stack effect’s total created airflow rate as in Section 4.2 by making several assumptions. The first assumption for the stack effect was that all openings with air flowing in are at a lower level of the void and that the ones with air flowing out are at a relative height z. Then for the wind effect’s n airflows we assumed that for all of them, the associated wind pressure coefficients are the same and we defined as $\Delta C_{p}=C_{p_{n_{i}}}-C_{p_{n_{j}}}$ ($C_{p_{n_{i}}}$ is the wind pressure coefficients of the incoming air openings *n_i_* and $C_{p_{n_{j}}}$ the wind pressure coefficients of the outgoing air openings *n_j_*). Then the total incoming air flow rate $F_{{\left(W+S\right)}_{sum}}$ in (32) becomes:

(33) $$F_{{\left(W+S\right)}_{sum}}\approx \underset{n}{\sum }\left(C_{d_{n}}A_{n}\sqrt{\Delta C_{p}{\left(C_{R}v_{ref}\right)}^{2}+2\frac{\left(T_{c}-T_{f}\right)gz}{T_{f}+273}}\right)\;\;\;\;\;\;\;\;\;\;=\underset{n}{\sum }\left(C_{d_{n}}A_{n}\right)\sqrt{\Delta C_{p}{\left(C_{R}v_{ref}\right)}^{2}+2\frac{\left(T_{c}-T_{f}\right)gz}{T_{f}+273}}$$

$C_{d_{n}}$ and $A_{n}$ are the discharge coefficient and the area respectively for each pair of openings. Then, further approximating $F_{{\left(W+S\right)}_{sum}}$ we assumed that cavities, even those of large size (50 m^3^), have height lower than 2–3 m, and T_c_ is uniform inside the cavity and doesn’t change. Also, we assumed that the average value of the discharge coefficients $C_{d_{n}}$ for all openings is 0.61 and the total area of all openings is *A_sum_*, thus $\underset{n}{\sum }\left(C_{d_{n}}A_{n}\right)\approx 0.61A_{sum}$. Then the approximated the total incoming air flow rate in (21) becomes:

(34) $$F_{{\left(W+S\right)}_{sum}}\approx 0.61A_{sum}\sqrt{\Delta C_{p}{\left(C_{R}v_{ref}\right)}^{2}+2\frac{\left(T_{c}-T_{f}\right)gz}{T_{f}+273}}$$

Obviously, in the rare case that *T_f_* > *T_C_*_,_ the outgoing air flow rate from the void it holds that:

(35) $$F_{{\left(W+S\right)}_{sum}}\approx 0.61A_{sum}\sqrt{\Delta C_{p}{\left(C_{R}v_{ref}\right)}^{2}+2\frac{\left(T_{f}-T_{c}\right)gz}{T_{c}+273}}$$

In this rare case of an “inverse” stack effect, comparing (34) and (35), it is evident that the incoming fresh airflow rate in the void decreases. In any other situation, the performance of the system with gas sensors deteriorates since the incoming fresh air flow rate in the void increases because of the combination of wind and stack effect.

Inequalities (12) and (13) in Section 3.3 revealed that the operational limits of the system with gas sensors depend on the incoming fresh air flow rate $F_{{\left(W+S\right)}_{sum}}$ in the void. In the case that the system with gas sensors could estimate $F_{{\left(W+S\right)}_{sum}}$ by taking into account weather data and the rubble’s characteristics (openings’ sizes), then the capability level for real time victim localization could be estimated as well.

Applying (34) for a void with z=2 m, a 5 °C temperature difference, for large openings on the front area of a cavity, in an urban/suburban terrain, and for various wind speeds $v_{z}$, for both minimum and maximum wind pressure coefficients differences $\Delta C_{p}$ (i.e., $\Delta C_{p_{min}}=0.44$, and $\Delta C_{p_{max}}=1.51$ respectively), in Table 8 the maximum total possible area of a large opening on the front of a void is estimated so that it is possible for either an adult male or a 3-year-old child to be detected by either an CO_2_ or an O_2_ sensor in the detection system.

Commenting on the results presented in Table 8 concerning the detection capabilities of the system with gas sensors when both wind and stack effects are acting concurrently, it has been shown that for the best-case scenarios with a minimum $\Delta C_{p}$ ($\Delta C_{p}=0.44$), the CO_2_ sensor will detect an adult human even when $v_{z}=10\;m/s$ with a front opening less than 30 cm x 30 cm and to detect a 3-year-old child, the opening must be less than 20 cm × 20 cm. For the worst-case scenarios of $\Delta C_{p}$ ($\Delta C_{p}=1.51$), the CO_2_ sensor will detect an adult man, when $v_{z}=10\;m/s$, with a front opening of less than 20 cm × 20 cm. For a 3-year-old child, the opening must be less than 10 cm × 10 cm. Similarly, the results for the O_2_ sensor detection capabilities, show that for the best-case scenarios when $v_{z}=10\;m/s$, an adult human will be detected when the front opening is less than 20 cm × 20 cm and to detect a 3-year-old child the opening must be less than 12 cm × 12 cm. For the worst-case scenario, the O_2_ sensor will detect an adult man, when $v_{z}=10\;m/s$, with a front opening of less than 11 cm × 11 cm. For a 3-year-old child, the opening has to be less than 6 cm × 6 cm.

From the analysis carried out in this section of the paper, a methodology can be followed to estimate the capability of a system with gas sensors for real time victim localization.

Generalizing (12) and (13) we get:

(36) $$\frac{F_{{\left(W+S\right)}_{sum}}C_{({\mathrm{CO}}_{2})f}+F_{e}C_{({\mathrm{CO}}_{2})e}}{F_{{\left(W+S\right)}_{sum}}+F_{e}}\geq \left(\bar{C_{({\mathrm{CO}}_{2})f}}+2stdev_{\bar{C_{({\mathrm{CO}}_{2})f}}}\right)$$

(37) $$\frac{F_{{\left(W+S\right)}_{sum}}C_{(O_{2})f}+F_{e}C_{(O_{2})e}}{F_{{\left(W+S\right)}_{sum}}+F_{e}}\leq \left(\bar{C_{(O_{2})f}}-2stdev_{\bar{C_{(O_{2})f}}}\right)$$

$stdev_{\bar{C_{({\mathrm{CO}}_{2})f}}}$ and $stdev_{\bar{C_{(O_{2})f}}}$ is the standard deviation values that result during calibration outside the rubble heap.

The proposed methodology is: first, to measure the concentrations of O_2_ and CO_2_ in the fresh air just outside the rubble before the search and estimate the standard deviation of each of the concentrations. Then, (34) is applied, by using the weather data (wind speed), and the estimated area of the openings (*A_sum_*), and appraising the maximum expected airflow rate $F_{{\left(W+S\right)}_{sum}}$ (using $\Delta C_{p_{max}}$). Next, the $F_{{\left(W+S\right)}_{sum}}$ is applied to (36) and (37) and the minimum *F_e_* that can be detected (actually the size of the victim) is estimated. The minimum detectable *F_e_* can finally be presented to the USaR operator as the estimated capability level for the performance of the system with gas sensors in real time victim localization. This will enable operators to decide if it is reliable enough to use the system and to prioritize the available search tools, thus not losing valuable time that may cost human lives. Testing and validation of the results of the analysis in this subsection are presented in Section 6 of this paper. The examined model is considered to be adequate for its intended purpose. This is because all other cases, with multiple cavities in series or multiple air interfaces, can be reduced to an equivalent of the described model. It is considered the worst-case scenario from a concentration detection perspective because it exhibits the most air exchange. Regarding the time required to reach equilibrium, as the number of interconnected cavities increase, the equivalent volume increases which affects the equilibrium time.

## 5. Gas Sensor Based Mobile Detection System

**Figure 1:**
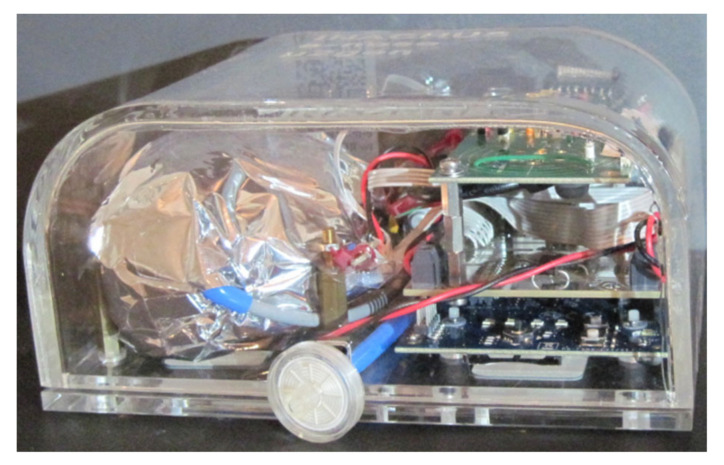
The detector device. On the left is the sensor cluster and on the right are the readout and control electronics. An air interface filter can be seen in the center of the image.

The system utilized for the experiments has been presented in [30]. The detector device has a cluster of gas and temperature sensors enclosed in an air manifold. The gas sensors are heated to a constant temperature in order to hinder humidity condensation. Because of its operation in harsh environments, hydrophobic air filters protect the system’s input and output air interfaces. A diaphragm air pump is used to force air through the system and facilitate a continuous air sampling scheme. The system with gas sensors also has readout, driving and control electronics, as well as a suitable enclosure for the operating environment. Sensor measurements are transmitted over a communication bus (Ethernet or USB) to a personal computer, such as a laptop, for processing and presentation in a user interface application. This application also facilitates the control of the detector device for preparation of deployment or servicing, such as sensor calibration. The detector device is illustrated in Figure 1.

The CO_2_ sensor is the CO2F-W and the O_2_ sensor is the LuminOx (LOX-02) from SST Sensing Ltd. They require periodic calibration to adhere to their error specification. Typical gas sensors exhibit a linear response to gas concentration changes. To correct the error, the response curve (line) must be corrected by modifying the response line’s gain, offset, or both. For the purpose of this work, a high accuracy of the gas concentration readings is not required, especially at larger concentration differentiations. This allows for the assumption that there is only an offset error which can be corrected by using measurements acquired at one known gas concentration, that of fresh air. Even if there is gain error, by selecting the calibration point where it is more critical, the error is reduced where it matters the most. For commercial gas detectors calibration must be performed on the device in real time. In the presented work, the calibration is performed offline after the acquisition of the measurements which include measurements of fresh clean air. This is single point calibration. No extra corrections are performed. For human detection, the absolute value of CO_2_ is not critical. A relative/comparative change of CO_2_ is required, and it is assumed that the reference measurement is sampled at the search location (same elevation), just before deployment. Also, variations of barometric pressure due to weather changes are insignificant (at least for the selected sensor). The O_2_ sensor is pressure sensitive but is pressure compensated because it includes a barometric pressure sensor. However, the pressure sensor introduces extra error in the O_2_% reading. Therefore, the sensor pump speed/flow is constant, and all valid calibration and measurements are taken when it is pumping and all filters are installed, so that a known error can be eliminated. The tested system also included a humidity sensor and two temperature sensors, one for sensor temperature and one for external temperature. The O_2_ measurements which are affected by humidity variations are not corrected for H_2_O dilution. This is because for human detection purposes, the dilution is caused by human presence and is a natural “amplification” of the measurement.

Human detection is achieved when places are detected where the local minimum of O_2_ and local maximum of CO_2_ concentrations exist while scanning with the air sampling port or moving the device. This concentration variation is calculated based on a reference reading made before starting the USaR operations outside of the rubble. Nevertheless, weather conditions presented in Section 4 may not allow human detection if (36) and (37) are not true. Thus, USaR teams should first evaluate the capability level of the system as described in Section 4.3. Equations (8) and (9) use 2 × SD, not standard practice 3 × SD for determining the noise floor. This design decision was made so the search for human presence could be assisted with a “third state”. It can be achieved by the non-latching output of the system, the relatively fast sampling rate and the system’s output refresh frequency; Transitioning from one state to the other helps determine approaching or moving away from a potential victim occupied cavity. An increasing frequency of “false” positives indicates approaching a point of interest.

## 6. Tests for the Verification of the Theoretical Analysis for the Performance of a System with Gas Sensors

The developed prototype system in [30] implemented the complete victim localization functionality, namely CO_2_, and O_2_ gas sensors, and it was used in the tests described in this paper to verify the theoretical analysis presented in Section 3 and Section 4. For a stand-alone system with gas sensors device, a threshold dictating human presence cannot be determined when the ventilation parameters and the number and kind of victims are unknown. Normally, observing the trends of sensor outputs helps in this direction by showing if the sensors’ output is increasing or decreasing), and the operator can infer that system’s air input, is getting closer to or is moving away from a victim. On approaching a victim, normally, the CO_2_ increases and the O_2_ decreases. The peak detection output shows the operator that while moving, the system’s air input passed by a possibly important location and he might have to backtrack and investigate in more detail. After providing information about the weather data and the estimated area of the openings of a void, it is highly important then that the USaR teams are informed by the system with gas sensors about its capability level of detecting a human victim.

The performance analysis and the theoretical approximations made in Section 3 and Section 4 were verified with a series of experiments which are presented in this section, using the system with gas sensors prototype. The main objective of the tests was to determine the detection capability of the system in controlled ventilation conditions. For this reason, the detector device was tested in various confined spaces with actual human subjects. Ethical considerations were taken into account during the design and planning of the experiments with the main focus on not putting the test subject (one or two of the design engineers), in hazardous and frustrating conditions. For these reasons there was always a ventilation opening adequate for maintaining non-hazardous air conditions inside the confined space, the test subjects were allowed to stop the experiment at any moment and exit the void by their own means, they were always supervised, seated and allowed to work in the confined space on a laptop, or tablet, or smart phone, under good lighting and with a view of the outside. The three tests were carried out in an office room, an office storage cupboard and an outdoor guard post. The office room test was planned for testing the detector device in an environment similar to a large cavity. The second test with an office storage cupboard emulated a small cavity deep inside the rubble that has limited capabilities of air refreshment. The outdoor guard post test targeted a relatively medium sized cavity (maximum capacity 2–3 people). All the tests had an initial phase (called Part I), that was required for the “calibration” of the device’s sensors by measuring the fresh atmospheric air concentrations of CO_2_ and O_2_ and by estimating the corresponding sensors’ noise level under the specific conditions of each of the tests. The system with gas sensors sampled the air very near to the enclosed space where the test took place (room, cupboard and guard post). The measurements for all phases of each of the tests are presented in graphs (one graph for each test), including the measurements of both sensors. The horizontal axis shows the time of day (hh:mm), during the entire test and the green vertical double lines separate the parts of each test scenario. The red line on the graphs represents the oxygen measurements (right vertical axis) and the light blue line represents the carbon dioxide measurements (left vertical axis). The horizontal dotted lines are the decision thresholds/limits of the sensors of the system with gas sensors used here in all three tests. These limits are calculated by using Equations (8) and (9) (presented in Section 3.2), the estimated mean values and standard deviations from the measurements of the fresh air concentrations of CO_2_ and O_2_, carried out in part I of each test. Concentration measurements may indicate human presence (entrapped victims in a cavity) with enough certainty and without creating false alarms to the rescue teams, only if they are over or below these limits (CO_2_ and O_2_ measurements respectively). The orange line represents the oxygen concentration limit by applying (9), and the light blue line similarly represents the CO_2_ sensor carbon dioxide concentration limit by applying (8). Part II of the tests verifies Equation (6) presented in Section 2 and the equilibrium time presented in Section 3.4 by limiting, as much as possible, the fresh air flow rate simulating an ideal case of a poorly ventilated cavity in all the tests. During part III of the tests, the ventilation of the simulated “cavity” was maximized to “reset” the concentrations to those of fresh atmospheric air. During part IV of the tests, the openings of the setup were decreased gradually until the estimated/expected fresh air flow rate, according to Equation (34), reached the calculated concentration detection threshold of the carbon dioxide sensor given by (8) and (36). Thus, all three equations/inequalities are verified. Then in part V the openings of the test setup were further decreased very slowly to an estimated fresh air flow rate that allows the concentration detection limits of the O_2_ sensor calculated in Equations (9) and (37) to also detect the human presence and verify those two inequalities as well. In all the three tests, during each part, the device’s sensors were sampled every 1 s. However, in the graphs presenting the measurements only the measurements taken every 1 min are plotted as this was considered to be appropriate for the chosen scenarios in order to verify the theoretical calculations about the performance of a system with gas sensors in USaR operations.

### 6.1. Room Tests

The first test took place in office premises, in a room of suitable dimensions (3.2 m 3.2 m × 5.5 m × 2.7 m), emulating a large cavity. It is expected that few cavities in real life scenarios will be larger than such a room of approximately 47.5 m^3^ in volume. Additionally, the immediate availability of this specific room rendered it a frequent testing “apparatus”. This office room allowed us to perform frequent tests during the development cycle of the system with gas sensors, to verify the validity of the theoretical results and identify potential discrepancies in various cases of fresh air circulation in a large room and to check the system’s gas sensors outputs. The openings of the room (an interior door 1.89 m^2^, and an outdoor south facing window with a maximum opening of 1 m^2^ and a minimum of 0.005 m^2^), allowed us to control the fresh air circulation during this first test. An additional aim of this test was to verify the time for equilibrium, according to the exhaust air flow rate, for a large volume void such as this room.

**Figure 2:**
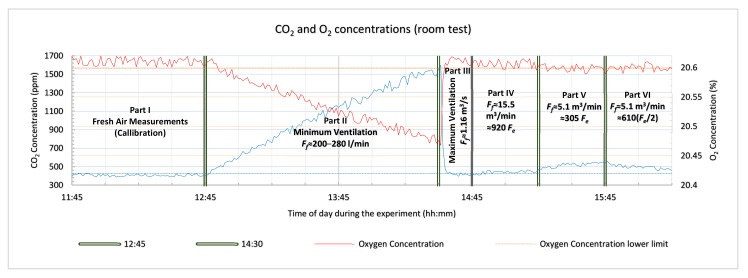
Room with two people test measurements.

When the test started at 11:45 the air in the room was refreshed by leaving the window and the door wide open for 60 min, to let fresh air in and minimize the effect of the exhaled human breath from within the room. During part I of the test (11:45–12:45), “calibration” of the system took place by sampling the outdoor air just outside the outdoor window of the room. As may be seen in Figure 2, the system with gas sensors revealed a mean concentration value for CO_2_ in the fresh atmospheric air of $\bar{C_{({\mathrm{CO}}_{2})f}}=405\;\mathrm{ppm}$ (left vertical axis) and for O_2_ $\bar{C_{(O_{2})f}}=20.61\%$ (right vertical axis) with standard deviations $stdev_{\bar{C_{({\mathrm{CO}}_{2})f}}}=12.31\;\mathrm{ppm}$ and $stdev_{\bar{C_{(O_{2})f}}}=0.0057\%$ respectively. By applying these values in Equations (36) and (37) and assuming that the concentrations in the exhaled breath are for CO_2_ 40000 ppm (the minimum [7,8,9,10]) and the maximum in O_2_ 16% [7,8,9,10], results show that the fresh air flow rate should be *F_f_* > ≈ 956 *F_e_* for it to be possible for the CO_2_ sensor to detect the human presence in the room and *F_f_* < ≈ 328 *F_e_* should hold for it to be possible for the O_2_ sensor to detect human presence.

Part II of the experiment started at 12:45 when we adjusted the window opening to just 0.5 cm wide, allowing fresh air to enter through an area of just 50 cm^2^ and 2 adult male persons entered the room. The large volume of the room would take a long time for the actual air mixture to change and reach equilibrium concentrations and for that reason it was convenient that two people were working in the room. Weather data for the day of the experiment showed a SE wind of $v_{ref}$=10 km/h and the outdoor temperature was measured $T_{f}\;$=20 °C. At the beginning of the experiment the temperature in the room was *T_c_ = T_f_ =* 20 °C, but slowly *T_c_* started to rise during part II of the test and after an hour it had risen to 25 °C. By applying (34) ($A_{sum}\;$= 0.005 m^2^ g = 9.81 m/s^2^, z = 2.7 m, $C_{R}\;$= 0.35, $v_{ref\;}$≈ 2.78 m/s, $\Delta C_{p}\;$= 0.44–1.51, $T_{f}\;$= 20 °C, and $T_{c}\;$= 25 °C), the expected fresh air flow rate was estimated to be *F_f_* ≈ 200–280 L/min. We assumed, according to Table 2, that the exhaled airflow rate for the 2 adult males would have an average value of *F_e_* = 16.8 L/min. Then it was calculated according to Section 3.4 and by using Equation (6) that the expected equilibrium concentration would be reached in 17.5 h and would be on average for CO_2_ 3335 ppm and for O_2_ 20.27%. We decided to limit the duration of part II of this test to 14:30 (105 min duration), and according to our calculations, we could thus reach a concentration ranging between 1593–2650 ppm for CO_2_ and 20.47–20.48% for oxygen. The actual measurements taken at the end of part II were 1590 ppm for CO_2_ and 20.47% for oxygen, (Figure 2), verifying the theoretical expected values.

Part III of the first test was planned to last for 15 min with the openings of the room at their maximum. The expected airflow rate on average 1.16 m^3^/s completely cleaned the indoor air, and the concentrations became the same as they were for the fresh atmospheric air.

Part IV of the test started at 14:45 and lasted 30 min. We closed the window halfway at 50 cm letting an opening with an area of $A_{sum}\;$= 0.5 m^2^. At the beginning of this part of the test the temperature in the room was measured and was found to be *T_c_ = T_f_* = 20.5 °C, but slowly *T_c_* started to rise and at the end of part IV of the first test it had risen to *T_c_* = 21.5 °C. By applying (34) ($A_{sum}\;$= 0.5 m^2^, g = 9.81 m/s^2^, z = 2.7 m, $C_{R}\;$= 0.35, $v_{ref}\;$≈ 0.83 m/s, $\Delta C_{p}\;$= 0.44–1.51, $T_{f\;}$= 20.5 °C, and $T_{c}\;$= 21.5 °C), we calculated that the expected fresh air flow rate at that point would be about 15.5 m^3^/min ie *F_f_* ≈ 920 *F_e_* and, according to the theoretical expectations from Equation (36) it required *F_f_ ≤* 956 *F_e_*, the CO_2_ sensor should identify human presence. In Figure 2, it can be seen that the CO_2_ sensor is clearly differentiated beyond its upper limit, indicating a human presence as expected theoretically from (36), with a value of 444 ppm. The oxygen sensor is within its noise zone with an average value of 20.6%, near the measured fresh air O_2_ concentration (20.61%).

Part V started at 15:15 and the duration was again 30 min. We continued by closing the window to a 15 cm opening, with an area of $A_{sum}\;$= 0.15 m^2^. At the beginning of the experiment the temperature in the room was measured and was found to be *T_c_* = 21.5 °C, and slowly started to rise and at the end of part V of the first test it had risen to 22.5 °C. By applying (34) ($A_{sum\;}$= 0.15 m^2^ g = 9.81 m/s^2^, z = 2.7 m, $C_{R}\;$= 0.35, $v_{ref}\;$≈ 0.83 m/s, $\Delta C_{p}\;$= 0.44–1.51, $T_{f\;}$= 21 °C, and $T_{c\;}$= 22.5 °C), we calculated that the expected fresh air flow rate at that point would be about 5.1 m^3^/min ie *F_f_ ≈* 305 *F_e_* and according to the theoretical expectations from (36) and (37) (CO_2_ sensor *F_f_ ≤* 956 *F_e_* and for O_2_ sensor *F_f_ ≤* 328 *F_e_*), both CO_2_ and O_2_ sensors should identify human presence. In Figure 2, it can be seen that the CO_2_ sensor is more clearly differentiated beyond its upper limit indicating a human presence, as expected theoretically from (36), with a value of 520 ppm. The oxygen sensor also reveals a human presence, as expected theoretically from (37), with an average value of 20.58%.

This first test had an additional sixth phase. In this last part of the first test the ventilation remained the same, but one of the two people left the room and the exhaled air flow rate decreased to *F_e_* ≈ 8.4 L/min, the fresh air flow rate remained unchanged (*F_f_* = 5.1 m^3^/min), thus their relationship became *F_f_* ≈ 610 *F_e_*. In Figure 2, it may be seen that the CO_2_ sensor is still clearly differentiated further beyond its upper limit indicating human presence, as expected theoretically from (36), with a value of 471 ppm. The oxygen sensor no longer shows human presence, as expected theoretically from (37), since the average value was 20.60% ie within the noise zone of the specific sensor used in the system with gas sensors used for the measurements.

Conclusion 1: All parts of the first room test confirmed the theoretical expectations and verified the analysis presented in this paper.

### 6.2. Office Storage Cupboard Test

**Figure 3:**
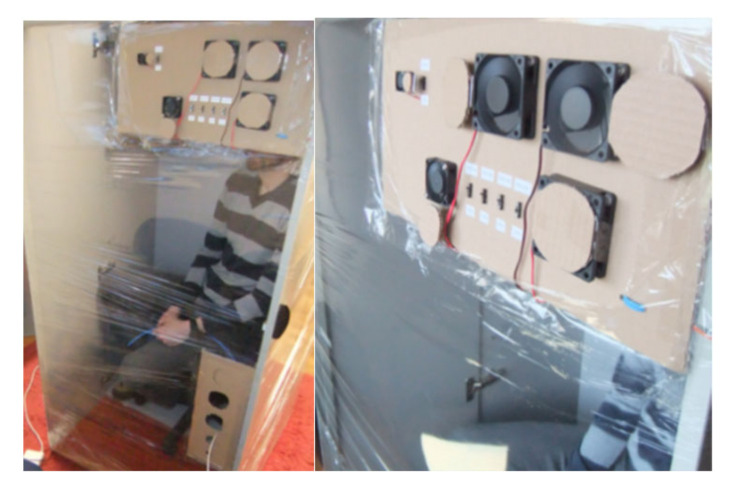
Left 2 fans ON~250 lpm Right 4 fans ON~1250 lpm.

For the second test, a confined space was created by sealing an office storage cupboard with food wrap and adding a cluster of fans with known airflow rate capability to adjust the incoming “fresh” airflow rate. Therefore, air conditions inside the cupboard were expected to simulate a rubble void with controlled fresh air flow by switching fans on and off and actually simulating a controlled wind effect. The office storage cupboard volume was extended by keeping the doors open with appropriate support (tube shown in Figure 3) and was then gradually sealed in order to control the fresh air entering the interior of the space and a procedure was adopted for closing the human subject inside the space, designed in such a way that there was no chance of suffocation. Also, the materials for creating the closed space and sealing the tube section could very easily be removed by hand. First, the human subject was placed in the space equipped with a paper cutter for an emergency exit. Next, the top half of the setup was sealed with film, including the top section, and then an opening of 20 cm × 20 cm was created in the installed film to host the fan setup. The installation and testing of the means of ventilation (fans), on the already installed film, followed. After installing and testing the fans through a panel in the top half of the test setup, the bottom half of the space was then also sealed with film without including the bottom section of the setup that was facing the floor in order to create a way-out for the incoming air from the fans. Thus, we created a sealed space that allowed airflow coming in from the fans and exiting from the bottom of the cupboard setup.

The measurements of the gas concentrations inside the simulated void were related to known/controlled ventilation values. During the test, the fans were switched ON or OFF to produce the required amounts of fresh air flow circulations during different parts of the test. Figure 3 illustrates this cupboard test setup, including the ventilation fan setup. On the top right of the cupboard space, the air fan cluster is positioned to control the “fresh” air flow rate. Each fan is controlled by an ON/OFF switch and the total flow is assumed to be the sum of the flow of each fan that was enabled. There were five fans in the cluster. Three of them had an airflow capability of, 2000 L/min, one of 1500 L/min and one of 75 L/min. The total air flow ideally was 7.575 m^3^/min.

**Figure 4:**
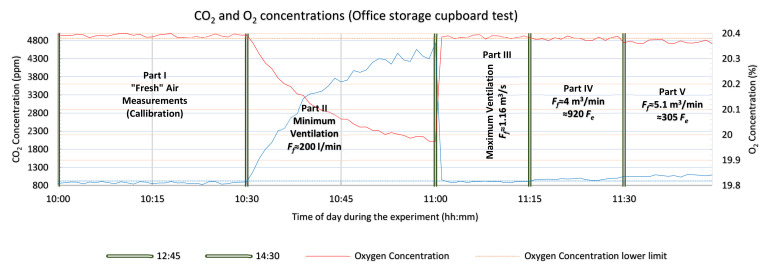
Extended office storage cupboard with one person test measurements.

The office storage cupboard was placed in a large corridor and the test measurements started at 10:00. During the preparation of the cupboard setup, “calibration” of the system with gas sensors took place by sampling the air in the corridor and the measured concentration mean values were $\bar{C_{({\mathrm{CO}}_{2})f}}=866\;\mathrm{ppm}$ and $\bar{C_{(O_{2})f}}=20.39\%$, with standard deviations $stdev_{\bar{C_{({\mathrm{CO}}_{2})f}}}=23.57\;\mathrm{ppm}$ and $stdev_{\bar{C_{(O_{2})f}}}=0.0051\%$. Applying these values in (36) and (37) and assuming that the concentrations in the exhaled breath are for CO_2_ 40,000 ppm (the minimum) and for O_2_ 16% (the maximum) [7,8,9,10], shows that that the fresh air flow rate should be *F_f_* ≤ 414 *F_e_* to be possible for the CO_2_ sensor to detect a human presence in the office storage cupboard and *F_f_* ≤ 215 *F_e_* should hold for it to be possible for the O_2_ sensor to detect a human presence. According to those measurements in part I the dotted orange line in Figure 4, represents the fresh air oxygen average concentration-2stdev (20.38%), measured by the O_2_ sensor and the light blue line similarly represents the fresh air carbon dioxide average concentration + 2stdev (912 ppm) for the CO_2_ sensor. The oxygen sensor measurements must be below this limit to detect entrapped victims in a cavity with enough certainty and without creating false alarms for the rescue teams, and similarly, the CO_2_ sensor measurements have to be below the estimated limit. Figure 4 displays the CO_2_ and O_2_ measurements with annotation regarding the phases of the experiment.

When the sealing of the cupboard was finished at 10:30, part II of the test started. The volume of the sealed created cavity was calculated as *V_c_* = 0.93 m^3^, and the exhaled air flow rate of the test subject (an adult male) *Fe* ≈ 8.4 L/min. It was calculated that with only the 75 L/min fan activated, according to Section 3.4 and by using Equation (6), the expected equilibrium concentration would be reached in 55 min and would be on average 4808 ppm for CO_2_ and 19.95% for O_2_. We decided to limit the duration of part II of this test to 30 min, and according to our estimations we could reach a concentration of about 4569 ppm for CO_2_ and 19.97% for oxygen. The actual measurements during part II of the test as may be seen in Figure 4 reached 4612 ppm for CO_2_ and 19.98% for oxygen, verifying the theoretical expected values.

Part III of the cupboard test was planned to last for 15 min with all the fans switched on (expected airflow rate on average 7.575 m^3^/min), to clean the air going into the cupboard enough and force the concentrations to become almost the same as the concentrations of the air in the corridor. The measured concentrations during part III reached 910 ppm for CO_2_ and 20.39% for oxygen, thus neither sensor detected a human presence because the high airflow rate forced them under and over their limits respectively (both calculated in part I of this test).

Part IV of the test followed and lasted for another 15 min. We left just two of the fans switched on to adjust the airflow at *F_f_* = 3500 L/min, thus, *F_f_* = 417 *F_e_* (*Fe* ≈ 8.4 L/min). According to our calculations in part I of this test the theoretical “demand” from (36), for the CO_2_ sensor to detect a human presence is *F_f_* ≤ 414 *F_e_*. For oxygen similarly from (37) and the part I measurements, *F_f_* ≤ 215 *F_e_* should hold. As can be seen in Figure 4, the carbon dioxide sensor conclusively detected a human presence (values about 912 ppm over the threshold- light blue dotted line). For the oxygen sensor, this was not possible since the result was still in the noise zone of 20.38%.

The last part of this test was part V with a duration again of 15 min. Just two of the fans were left switched on in order to adjust the airflow to *F_f_* = 1575 L/min, thus *F_f_* = 188 *F_e_* (*Fe* = 8.4 L/min). According to the calculations in part I of this test the theoretical “demand” from (37), for the O_2_ sensor to detect a human presence is that *F_f_* ≤ 215 *F_e_* should hold true. As can be seen in Figure 4, both the oxygen and, certainly, the carbon dioxide sensor obviously detected a human presence. The value of the O_2_ concentration was 20.38% under the threshold orange dotted line and the CO_2_, 1002 ppm, was over the threshold light blue dotted line.

Conclusion 2: The office storage cupboard test also fully confirmed the theoretical expectations and verified the analysis presented in this paper.

### 6.3. Outdoor Guard Post Test

The outdoor guard post with controlled openings (a door and three windows), allowed us to examine the influence of fresh airflow rate on concentration measurements according to the analysis presented in Section 2, Section 3 and Section 4.

**Figure 5:**
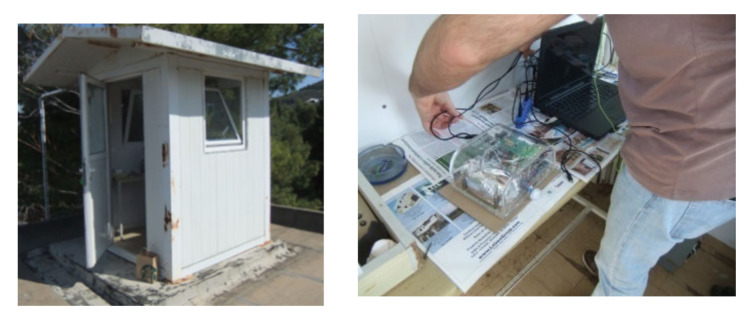
**Left**: guard post with door and windows. **Right**: detector device with the sampling tube and the entire measurement setup.

The photographs in Figure 5 illustrate the guard post test location. The dimensions of the guard post were 1.2 m × 1.2 m × 2.3 m (*V_c_* = 2.3 m^3^), simulating a medium-small cavity. The openings of the guard post were on the north side.

**Figure 6:**
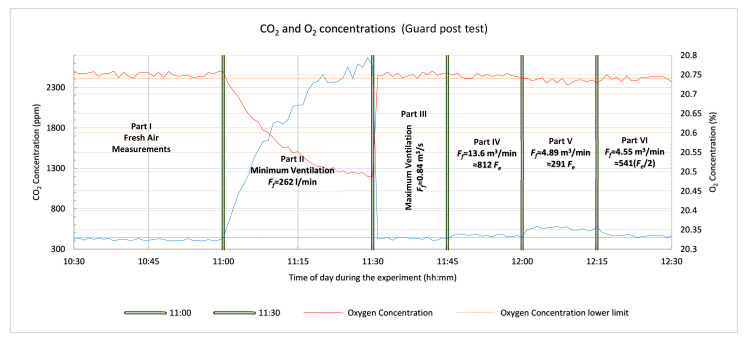
Guard post test measurements.

Part I: As in the last test, the “calibration session” commenced by taking measurements with the air sampling tube placed further from the openings of the guard post. The processed measurements yielded averaged concentrations for CO_2_ and O_2,_ $\bar{C_{({\mathrm{CO}}_{2})f}}=$ 418 ppm with a standard deviation of 12.22 and $\bar{C_{(O_{2})f}}=$ 20.75% with a standard deviation of 0.006 (Figure 6). At the same time the air inside the guard post was refreshed by leaving all the windows and the door wide open for 30 min, to let fresh air in and reset the concentrations within the room. By applying the values in Equations (36) and (37) and assuming that the concentrations of the exhaled breath are 40,000 ppm for CO_2_ (minimum) and for O_2_ 16% (maximum) [7,8,9,10], showing that the fresh air flow rate should be *F_f_ ≤* 808 *F_e_* for the CO_2_ sensor to detect a human presence in the room and *F_f_ ≤* 324 *F_e_* should hold true for the O_2_ sensor respectively.

Part II: This part of the test was performed with the door closed and with two human subjects inside the guard post (adult males with an expected average $F_{e}\approx 16.8\;L/\min$). At the end of part I, the air sampling tube was inserted into the guard post to start the measurements for part II. The outdoor temperature was 21 °C and the wind about 8 km/h, in a northerly direction (weather report on the day and at the time of the experiment). At the beginning of the experiment, the temperature in the interior of the guard post was *T_c_* = *T_f_*= 21 °C, but at the end it had risen to 23 °C. With the openings of the windows adjusted approximately to 100 cm^2^ (*A_i_* = 0.01 m^2^), and taking into account the wind and temperature data, (34), ($A_{sum}\;$= 0.01 m^2^ g = 9.81 m/s^2^, z = 0.7 m, $C_{R}\;$= 0.35, $v_{ref}\;$≈ 2.22 m/s, $\Delta C_{p}$= 0.44–1.51, $T_{f\;}$= 21 °C, and $T_{c\;}$= 23 °C) was applied, and an expected fresh air flow rate through the guard post of $F_{f}\approx 144-433\;L/\min$ and $F_{f}\approx 8.62-25.83\;F_{e}$ was estimated. Then, it was calculated according to Section 3.4 and by using Equation (6) that the expected equilibrium concentration would be reached in 37–102 min and would be, on average, 1895–4534 ppm for CO_2_ and for O_2_ 20.26–20.57%. It was decided to limit the duration of part II of this final test to 30 min duration, and according to our calculations, concentrations ranging between 1863–3570 ppm for CO_2_ and 20.36–20.58% for oxygen could be reached. After 30 min $C_{(CO_{2})c}$ reached a maximum of approximately 2604 ppm, while for O_2_ the concentration dropped to almost 20.49% as shown in Figure 6, which was in accordance with our theoretical expectations.

Part III: As in the first test there was a break of 15 min duration and the window openings of the guard post were open to their maximum (expected airflow rate on average 0.84 m^3^/min), which almost completely cleared the indoor air when the CO_2_ concentration was reduced to 433 ppm and the oxygen concentration became 20.75%, both concentrations reaching those of the fresh atmospheric air.

Part IV: This lasted for 15 min. The windows were closed leaving an opening with an area of $A_{sum}\;$= 0.6 m^2^. At the beginning of this part of the test the temperature in the guard post was measured and was found to be *T_c_* = *T_f_* = 21 °C, but it slowly started to rise and at the end of this part of the test it had risen to 21.5 °C. By applying (34) ($A_{sum}\;$= 0.6 m^2^, g = 9.81 m/s^2^, z = 0.7 m, $C_{R}\;$= 0.35, $v_{ref}\;$≈ 0.83 m/s, $\Delta C_{p}\;$= 0.44–1.51, $T_{f}\;$= 21 °C, and $T_{c}\;$= 21.5 °C), it was calculated that the expected fresh air flow rate at that point would be about 13.65 m^3^/min i.e., F_f_ ≈ 812 F_e_ and according to the theoretical expectations from (36) (*F_f_ ≤* 808 *F_e_*), the CO_2_ sensor should identify a human presence. In Figure 6 it can be seen that the CO_2_ sensor is clearly differentiated beyond its upper limit indicating a human presence, which was theoretically expected from (36), with a value of 462 ppm. The oxygen sensor is within its noise zone with an average value 20.75%, equal to the measured fresh air O_2_ concentration (20.75%).

Part V: This once again lasted for 15 min. The window was adjusted by leaving an opening with an area of $A_{sum}\;$= 0.20 m^2^. At the beginning of this part of the last test the temperature in the room was measured and was found to be *T_c_* = 21.5 °C, and slowly started to rise until at the end of part V of the first test it had risen to 22 °C. By applying (34) ($A_{sum}\;$= 0.20 m^2^ g = 9.81 m/s^2^, z = 0.7 m, $C_{R\;}$= 0.35, $v_{ref}\;$≈ 0.83 m/s, $\Delta C_{p}\;$= 0.44–1.51, $T_{f\;}$= 21 °C, and $T_{c}\;$= 22 °C), the expected fresh air flow rate at that point was calculated to be about 4.89 m^3^/min ie *F_f_ ≈* 291 *F_e_* and according to the theoretical expectations from (36) and (37) (*F_f_ ≤* 808 *F_e_* for the CO_2_ sensor and *F_f_ ≤* 324 *F_e_* for the O_2_ sensor), both the CO_2_ and O_2_ sensors should identify a human presence. In Figure 6, it can be seen that the CO_2_ sensor is more clearly differentiated beyond its upper limit indicating a human presence, as was expected theoretically from (36), with a value of 547 ppm. The oxygen sensor also reveals a human presence, which was expected theoretically from (37), with an average value 20.73%.

Part VI: This third and last test had a sixth phase. For this last part, the openings remained the same as in the previous part, but one of the two people inside the guard post left and the exhaled air flow rate decreased to *F_e_* ≈ 8.4 L/min. At the beginning of this part of the test, the temperature inside was measured and found to be *T_c_* = 22 °C, which then slowly started to fall and at the end, it had fallen to 21.5 °C. By applying (34) ($A_{sum}\;$= 0.2 m^2^ g = 9.81 m/s^2^, z = 0.7 m, $C_{R}\;$= 0.35, $v_{ref}\;$≈ 0.83 m/s, $\Delta C_{p}\;$= 0.44–1.51, $T_{f}\;$= 21 °C, and $T_{c}\;$= 21.5 °C), we calculated that the expected fresh air flow rate at that point would be about 4.55 m^3^/min ie *F_f_ ≈* 541 *F_e_* thus *F_f_ ≈* 542 *F_e_*. In Figure 6, it can be seen that the CO_2_ sensor is still clearly differentiated beyond its upper limit indicating a human presence, confirming the theory in (36), with a value of 464 ppm. The oxygen sensor does not however, show a human presence, which was expected from the theory in (37), since the average value was 20.74%, i.e., within the noise zone of the specific sensor used in the system with gas sensors used for these measurements.

Conclusion 3: This third and last test in the guard post confirmed the theoretical expectations and verified the analysis presented in this paper.

## 7. Conclusions

In this paper, the performance of a mobile system with gas sensors was studied, from a theoretical analysis perspective. The analysis was performed for a system with gas sensors that measures the atmospheric air gas components concentrations (CO_2_ and O_2_), inside a rubble void, where humans are entrapped, after a disastrous event that caused the collapsing of a building/construction. Firstly, the equations calculating the anticipated concentration of CO_2_ and O_2_ inside a void as a function of time were formulated. Then, the anticipated performance of such a system was examined considering various cases with parameters, such as the kind of entrapped humans in a void, the characteristics of the CO_2_ and O_2_ sensors and the anticipated noise floor at a typical detection scenario location, and the total area of a void’s apertures. Finally, the effect of the external weather conditions, namely the effect of the possible external wind, as well as the possible temperature differences between the void and the outdoor environment, were examined, as well as their influence on the performance of this detection system. In all cases, the expected levels of CO_2_ and O_2_ were calculated, based on rational assumptions for the parameters, in order to produce an approximation of the capability of human detection in a mobile gas sensor system, with sensors of a specific sensitivity and specificity, when searching for trapped humans inside the void of a rubble pile. Three verification tests confirmed our theoretical analysis (Section 6.1, Section 6.2 and Section 6.3).

The findings of this work quantify the influence of the studied parameters, concerning the concentration of CO_2_ and O_2_ inside a rubble void and estimate the expected human presence detection limits of a system with gas sensors with specific sensor characteristics. It was shown that the system with gas sensors can indeed detect human presence in a plethora of scenarios. It was also theoretically shown that the system with gas sensors is not the ideal human detection system and cannot detect human presence in all scenarios, but the basis for determining such cases was established.

As part of future work, this analysis can be extended to cover more target gases and sensors as the detector device evolves into an e-nose.

## Figures and Tables

**Table 1:** Analysis parameters.

Parameter	Symbol	Unit
Concentration of gas x in fresh atmosphairic air	*C_xf_*	Volume concentration
Concentration of gas x in exhaled air	*C_xe_*	Volume concentration
Concentration of gas x exiting cavity	*C_xc_*	Volume concentration
Concentration of gas x in inhaled air	*C_xi_*	Volume concentration
Air flow rate flowing into the cavity	*F_f_*	L/min
Exhaled breath flow rate	*F_e_*	L/min
Air flow rate flowing out the cavity	*F_c_*	L/min
Inhaled breath flow rate	*F_i_*	L/min
Air temperature inside the cavity	*T_c_*	°C
Air temperature external to the cavity	*T_f_*	°C

**Table 2:** Exhaled air flow rates for individuals at different ages and weights.

Description of Individual	Average Weight (kg)	Average Number of Breaths/min	Tidal Volume (L)	$\mathrm{Average}\;F_{e}\;(L/\min)$
adult male (35 yo, 25% percentile)	75	12–20	0.5	6.3–10.5
adult female (35 yo, 25% percentile)	57	12–20	0.4	4.7–7.8
child (10 years old)	33	15–20	0.2	3.0–4.0
child (3 years old)	14	20–30	0.1	2.0–3.0
baby (6 months old)	7.5	25–40	0.1	2.5–4.0
elderly male (>80 yo, 25% percentile)	72	10–18	0.5	5.3–9.5
elderly female (>80 yo, 25% percent.)	52	10–18	0.4	3.9–7.0

**Table 3:** Statistical data for noise of CO_2_ and O_2_ sensors. Historical data extended to last 1 h.

	CO_2_ (ppm)	O_2_ (%)
**mean**	406	20.720
**st.dev**	21.92	0.01
**st.dev (%)**	5.4	0.05

**Table 4:** Estimation of equilibrium time.

Description of Entrapped Human	$\mathrm{Average}\;F_{e}\;(L/\mathrm{Min})$	Cavity/Void Size m^3^	Average Time (Min) Needed for Detection by the CO_2_ Sensor $\left(F_{f}\leq 871.74\;F_{e}\right)$	Average Time (Min) Needed for Detection by Both Sensors CO_2_ & O_2_ $\left(F_{f}\geq 440.76\;F_{e}\right)$
adult male	6.3–10.5	52.5	41	71
elderly female	3.9–7.0		64	111
child	3.0–4.0		93	163
child (3 years old)	2.0–3.0		132	232
adult male	6.3–10.5	26.5	21	36
elderly female	3.9–7.0		32	57
child	3.0–4.0		47	82
child (3 years old)	2.0–3.0		67	117
adult male	6.3–10.5	10	8	14
elderly female	3.9–7.0		13	22
child	3.0–4.0		18	31
child (3 years old)	2.0–3.0		26	45

**Table 5:** Estimation of $\Delta {p_{W_{f}}}_{“i-j”}=P_{W}{{}_{f}}_{i}-P_{W}{{}_{f}}_{j}$ for urban/suburban rubble void.

Wind Speed *v*_*ref*_ (m/s)	Front Surface ${P_{W_{f}}}_{i}$ Range (Pa)	Other Surface ${P_{W_{f}}}_{j}$ Range (Pa)	Minimum $\Delta {p_{W_{f}}}_{“i-j”}$ (Pa)	Maximum $\Delta {p_{W_{f}}}_{“i-j”}$ (Pa)
0.25	0.0021–0.0202	−0.0001–−0.0222	0.0022	0.0424
1	0.0328–0.324	−0.0016–−0.3555	0.0344	0.6795
3	0.2953–2.9157	−0.0141–−3.1992	0.3094	6.1149
5	0.8202–8.0993	−0.0391–−8.8867	0.8593	16.986
7	1.6077–15.8746	−0.0766–−17.418	1.6843	33.2926
10	3.281–32.3972	−0.1562–−35.5469	3.4372	67.9441

**Table 6:** Estimation of maximum total possible area of large openings in voids (Urban/Suburban).

Wind Speed m/s	Description of Entrapped Human	*A*_*i*_ (m^2^) for Max. $\Delta {p_{W_{f}}}_{“i-j”}$ CO_2_ Sensor	*A*_*i*_ (m^2^) for Min. $\Delta {p_{W_{f}}}_{“i-j”}$ CO_2_ Sensor	*A*_*i*_ (m^2^) for Max. $\Delta {p_{W_{f}}}_{“i-j”}$ CO_2_ & O_2_ Sensors	*A*_*i*_ (m^2^) for Min. $\Delta p_{W_{f}}{}_{“i-j”}$ CO_2_ & O_2_ Sensors
0.25	child 3 y	0.2199	0.9653	0.1248	0.5480
	adult male	0.7476	3.2820	0.4244	1.8634
3	child 3 y	0.0183	0.0814	0.0104	0.0462
	adult male	0.0623	0.2768	0.0353	0.1571
5	child 3 y	0.0110	0.0488	0.0062	0.0277
	adult male	0.0374	0.1661	0.0212	0.0943
10	child 3 y	0.0055	0.0244	0.0031	0.0139
	adult male	0.0187	0.0830	0.0106	0.0471

**Table 7:** Estimation of maximum total possible area of openings in voids (Stack effect).

$T_{C}-T_{f}$ °C	Description of Entrapped Human	$A_{sum}$ (m^2^) CO_2_ & O_2_ Sensors *z* = 1 m	$A_{sum}$ (m^2^) CO_2_ & O_2_ Sensors *z* = 2 m	$A_{sum}$ (m^2^) CO_2_ & O_2_ Sensors *z* = 3 m
3	child 3 y	1.1664	0.8248	0.6734
	adult male	3.9658	2.8043	2.2897
5	child 3 y	0.9035	0.6389	0.5216
	adult male	3.0719	2.1722	1.7736
10	child 3 y	0.6389	0.4518	0.3689
	adult male	2.1722	1.5360	1.2541
15	child 3 y	0.5216	0.3689	0.3012
	adult male	1.7736	1.2541	1.0240

**Table 8:** Estimation of maximum total possible area of openings in voids (Wind and Stack effect, *z* = 2 m, $T_{f}-T_{C}=5\;$ °C.

$\mathrm{Wind}\;\mathrm{Speed}\;v_{ref}$ (m/s)	Description of Entrapped Human	$\Delta C_{p_{max}}=1.51$ CO_2_ Sensor Max. **A*_*i*_* (m^2^)	$\Delta C_{p_{min}}=0.44$ CO_2_ Sensor Max. **A*_*i*_* (m^2^)	$\Delta C_{p_{max}}=1.51$ O_2_ Sensor Max. **A*_*i*_* (m^2^)	$\Delta C_{p_{min}}=0.44$ O_2_ Sensor Max. **A*_*i*_* (m^2^)
0.25	child 3 y	0.0645	0.0673	0.0366	0.0382
	adult male	0.2194	0.2289	0.1245	0.1300
3	child 3 y	0.0177	0.0520	0.0100	0.0295
	adult male	0.0601	0.1766	0.0341	0.1003
5	child 3 y	0.0108	0.0396	0.0062	0.0225
	adult male	0.0369	0.1345	0.0209	0.0764
10	child 3 y	0.0055	0.0230	0.0031	0.0130
	adult male	0.0186	0.0781	0.0106	0.0443

## References

[1] Tran J., Ferworn A., Gerdzhev M., Ostrom D. Canine assisted robot deployment for urban search and rescue. Proceedings of the IEEE International Workshop on Safety Security and Rescue Robotics (SSRR).

[2] Huo R., Agapiou A., Bocos-Bintintan V., Brown L.J., Burns C., Creaser C.S., Devenport N.A., Gao-Lau B., Guallar-Hoyas C., Hildebrand L. (2011). The trapped human experiment. J. Breath Res..

[3] Butcher K., Craig B., Chartered E. (2015). Environmental Design: Guide A.

[4] Irving S., Ford B., Etheridge D. (2005). Natural Ventilation in Non-Domestic Buildings.

[5] Liddament Martin W. (1996). A Guide to Energy Efficient Ventilation.

[6] Etheridge D.W. (2004). Natural Ventilation through Large Openings—Measurements at Model Scale and Envelope Flow Theory. Int. J. Vent..

[7] Brimblecombe P. (1986). Air: Composition & Chemistry.

[8] Colinetlagneaux D., Troquet J. (1972). Development of Gaseous Composition of Exhaled Air in Man during Calm and Forced Breathing. Arch. Int. Physiol. Biochim..

[9] Saidel G.M., Lin J.S. (1986). Transport Abnormalities from Single-Breath Dynamics of Ar, CO_2_ and O_2_. Respir. Physiol..

[10] Shusterman A.A., Teige V.E., Turner A.J., Newman C., Kim J., Cohen R.C. (2016). The BErkeley Atmospheric CO_2_ Observation Network: Initial evaluation. Atmos. Chem. Phys..

[11] Aukburg S.J., Neufeld G.R., Levine S., Scherer P.W. (1985). Effective Diffusing Area (Eda) Derived from Single Breath O-2 and Co-2 Exhalation Curves Compared. Fed. Proc..

[12] Yamagishi H., Tohjima Y., Mukai H., Nojiri Y., Miyazaki C., Katsumata K. (2012). Observation of atmospheric oxygen/nitrogen ratio aboard a cargo ship using gas chromatography/thermal conductivity detector. J. Geophys. Res. Atmos..

[13] Badawy J., Nguyen O.K., Clark C., Halm E.A., Makam A.N. (2017). Is everyone really breathing 20 times a minute?. Assessing epidemiology and variation in recorded respiratory rate in hospitalised adults. BMJ Qual. Saf..

[14] Rodriguez-Molinero A., Narvaiza L., Ruiz J., Galvez-Barron C. (2013). Normal respiratory rate and peripheral blood oxygen saturation in the elderly population. J. Am. Geriatr. Soc..

[15] Fleming S., Thompson M., Stevens R., Heneghan C., Plüddemann A., Maconochie I., Tarassenko L., Mant D. (2011). Normal ranges of heart rate and respiratory rate in children from birth to 18 years of age: A systematic review of observational studies. Lancet.

[16] Ratjen F., Jensen R., Klingel M., McDonald R., Moore C., Benseler N., Wilson D., Stanojevic S. (2019). Effect of changes in tidal volume on multiple breath washout outcomes. PLoS ONE.

[17] DeBoer S.L. (2004). Emergency Newborn Care.

[18] Lindh W.Q., Pooler M., Tamparo C.D., Dahl B.M., Morris J. (2013). Delmar’s Comprehensive Medical Assisting: Administrative and Clinical Competencies.

[19] Yuan G., Drost N.A., McIvor R.A. (2013). Respiratory rate and breathing pattern. Mcmaster Univ. Med. J..

[20] Anyfantis A., Blionas S. Design and development of a mobile e-nose platform for real time victim localization in confined spaces during USaR operations. Proceedings of the 2020 IEEE International Instrumentation and Measurement Technology Conference (I2MTC).

[21] Anyfantis A., Blionas S. Indoor air quality monitoring sensors for the design of a simple, low cost, mobile e-nose for real time victim localization. Proceedings of the PACET 2019.

[22] Davis N., Badger J., Hahmann A.N., Hansen B.O., Olsen B.T., Mortensen N.G., Heathfield D., Onninen M., Lacave G.L.O. (2019). Global Wind Atlas v3.

[23] Miller R.W. (1996). Flow Measurement Engineering Handbook.

[24] Qu M., Wan J., Hao X. (2014). Analysis of diurnal air temperature range change in the continental United States. Weather Clim. Extrem..

[25] Wang K., Ye H., Chen F., Xiong Y., Wang C. (2012). Urbanization effect on the diurnal temperature range: Different roles under solar dimming and brightening. J. Clim..

[26] Thomas S., Ravishankaran S., Justin N.J.A., Asokan A., Kalsingh T.M.J., Mathai M.T., Valecha N., Montgomery J., Thomas M.B., Eapen A. (2018). Microclimate variables of the ambient environment deliver the actual estimates of the extrinsic incubation period of Plasmodium vivax and Plasmodium falciparum: A study from a malaria-endemic urban setting, Chennai in India. Malar J..

[27] Cator L.J., Thomas S., Paaijmans K.P., Ravishankaran S., Justin J.A., Mathai M.T., Read A.F., Thomas M.B., Eapen A. (2013). Characterizing microclimate in urban malaria transmission settings: A case study from Chennai, India. Malar J..

[28] Franck U., Krüger M., Schwarz N., Grossmann K., Röder S., Schlink U. (2013). Heat stress in urban areas: Indoor and outdoor temperatures in different urban structure types and subjectively reported well-being during a heat wave in the city of Leipzig. Meteorol. Z..

[29] Herman I. (2007). Physics of the Human Body.

[30] Anyfantis A., Blionas S. (2020). Proof of concept apparatus for the design of a simple, low cost, mobile e-nose for real-time victim localization (human presence) based on indoor air quality monitoring sensors. Sens. Bio-Sens. Res..

